# Cytoplasmic flows in starfish oocytes are fully determined by cortical contractions

**DOI:** 10.1371/journal.pcbi.1006588

**Published:** 2018-11-15

**Authors:** Nils Klughammer, Johanna Bischof, Nikolas D. Schnellbächer, Andrea Callegari, Péter Lénárt, Ulrich S. Schwarz

**Affiliations:** 1 Institute for Theoretical Physics and BioQuant, Heidelberg University, Heidelberg, Germany; 2 Cell Biology and Biophysics Unit, European Molecular Biology Laboratory (EMBL), Heidelberg, Germany; Northeastern University, UNITED STATES

## Abstract

Cytoplasmic flows are an ubiquitous feature of biological systems, in particular in large cells, such as oocytes and eggs in early animal development. Here we show that cytoplasmic flows in starfish oocytes, which can be imaged well with transmission light microscopy, are fully determined by the cortical dynamics during surface contraction waves. We first show that the dynamics of the oocyte surface is highly symmetric around the animal-vegetal axis. We then mathematically solve the Stokes equation for flows inside a deforming sphere using the measured surface displacements as boundary conditions. Our theoretical predictions agree very well with the intracellular flows quantified by particle image velocimetry, proving that during this stage the starfish cytoplasm behaves as a simple Newtonian fluid on the micrometer scale. We calculate the pressure field inside the oocyte and find that its gradient is too small as to explain polar body extrusion, in contrast to earlier suggestions. Myosin II inhibition by blebbistatin confirms this conclusion, because it diminishes cell shape changes and hydrodynamic flow, but does not abolish polar body formation.

## Author summary

As already noted by Aristotle, life is motion. On the molecular scale, thermal motion leads to diffusive transport. On cellular scales, however, diffusion starts to become inefficient, due to the general property of random walks that their spatial excursions grow less than linear with time. Therefore more directed transport processes are needed on cellular scales, including transport by molecular motors or by hydrodynamic flows. This is especially true for oocytes and eggs in early animal development, which often have to be large in order to store sufficient amounts of nutrients. Here we use starfish oocytes as a convenient model system to investigate the nature and function of cytoplasmic flows in early development. These cells are very large and optically transparent, and therefore ideal for live cell imaging that here we combine with image processing and mathematical modelling. This approach allows us to demonstrate that the experimentally observed cytoplasmic flows during early development are a direct consequence of surface contraction waves that deform the soft and contractile eggs. Additionally we show that despite its microscopic complexity, the cytoplasm behaves like a Newtonian fluid on the cellular scale. Our findings impose strong physical limits on the potential biological function of these flows and suggest that also other cellular systems that are soft and contractile might experience large cytoplasmic flows upon cell shape changes, for example during cell migration or division.

## Introduction

The three most important cellular processes that drive animal development are cell division, cell shape changes and cell migration, which are all mediated by the cytoskeleton [1, 2]. Strikingly, cytoskeletal regulation during early animal development often takes the form of mechanical-biochemical waves or pulses, for example in axolotl [3], starfish [4], *Xenopus* [5], *Drosophila* [6–8] and mouse [9]. The biological function of such waves ranges from global coordination of cell division to localising cellular structures or molecular components [10–12].

In principle, it is expected that forces generated by the cytoskeleton lead to intracellular flows. Indeed cytoplasmic flows have been shown to have important functions in development, and have been proposed to mediate intracellular transport [13–15], intracellular mixing [16], force distribution [17, 18] and pattern formation [19]. However, the relation between wave-like cell shape changes and cytoplasmic flows has rarely been investigated in quantitative detail in a developmental model system, mainly due to experimental technical limitations.

Starfish oocytes are an ideal model system to investigate this relation because they are available in large numbers, they are large (diameter 180 μm), transparent (unlike most vertebrate eggs e.g. *Xenopus* or zebrafish) and are not enclosed by a stiff shell (like eggs of *Drosophila* or *C. elegans*). During early development, several stereotypical surface contraction waves (SCWs) run over the cell surface, leading to large surface deformations and intracellular flows [4, 20, 21]. However, despite a long tradition of studying them, it is unknown how exactly the surface deformations and the internal flows are related and what their biological functions are.

In detail, it is not clear if the internal flows are a direct physical consequence of the cell shape changes or if the cytoplasmic flows are also driven by forces generated in the cytoplasm. For instance it has been shown that flows in *Drosophila* oocytes are driven by cytoplasmic microtubule networks [14] and that flows in *C. elegans* embryos are driven by cortical tension gradients along the AP-axis [22]. However, in terms of the physical properties, a key difference is that *Drosophila* and *C. elegans* develop in a stiff shell, which is typically not the case for deuterostome species that include vertebrates and also the echinoderm starfish. Such eggs without a shell, like starfish, have the possibility to drive large scale cytoplasmic flows by normal deformations of their soft cortex. Indeed, comparable cortical contractions have been observed in vertebrates like frog or mouse, clearly demonstrating the general importance of this process.

For different species SCWs have been suggested to be involved in polar body extrusion, but the physical basis has not been tested quantitatively. This is particularly true for starfish, for which it has been suggested early on that the cytoplasmic flow is causal for the extrusion of the polar body following the SCW [4], but quantitative evidence for this suggestion has been missing. Although it has later been demonstrated that polar body formation is not directly affected by forced changes in cytoplasmic flows [23], the notion that the combination of hydrodynamic flow and local cortical weakening drives polar body extrusion is still present in the literature [24, 25]. We recently showed that the polar body is generated even if the SCW is strongly reduced by myosin II inhibition [21], but our earlier study did not address hydrodynamic flows.

To study the physical relation between surface deformation and cytoplasmic flow in soft oocytes as well as its biological consequences for polar body extrusion, here we combine live cell imaging, quantitative image processing and biophysical modelling in starfish. During maturation of starfish oocytes there are two meiotic divisions, each leading to the formation of one polar body. Each is preceded by a surface contraction wave leading to surface deformations and internal hydrodynamic flow. In this study we focus on the most prominent first SCW that is associated with meiosis I. It takes about seven minutes to run over the surface of the oocyte of 180 μm diameter. Because the focus of our work is on hydrodynamic flow, we will use transmission light microscopy, which in starfish oocytes gives excellent contrast due to the presence of intracellular yolk particles and thus can be used to reconstruct hydrodynamic flow using particle image velocimetry (PIV). We present a complete quantitative analysis of cell shape changes and hydrodynamic flows that demonstrate that the SCWs are the direct physical cause for the observed cytoplasmic flows. Thus no other mechanism is required to explain cytoplasmic flows during the SCW. Using a contraction model, we can not only study the large normal surface changes, but also the small tangential components. As an immediate consequence of our analytical treatment, we now can calculate the internal pressure field, that is not directly accessible experimentally [26] and has been obtained in similar systems before only in a numerical manner [17, 22, 27, 28]. Our analytical calculations are ideal to quickly process large data sets and show that the cytoplasmic flows caused by the SCWs are not sufficient to explain polar body extrusion, in contrast to earlier suggestions [4]. We finally show that inhibition of myosin II activity by blebbistatin does strongly reduce both cell shape changes and hydrodynamic flow, but not polar body formation, which seems to be caused by myosin II-independent local polymerisation of actin.

We start by imaging the full three-dimensional (3D) shape of freely floating oocytes labelled with fluorescent dextran, in order to demonstrate that the SCW appears to be rotational symmetric around the animal-vegetal (AV) axis. Based on this symmetry, we then switch to two-dimensional (2D) slices containing the AV-axis acquired with transmission light microscopy for oocytes in imaging chambers. This approach allows us to simultaneously quantify cell shape changes and cytoplasmic flows. Using a Fourier decomposition of the contour, we confirm that the SCW is highly symmetric in the 2D imaging plane regarding the AV-axis. We then use differential geometry for axisymmetric shapes to calculate previously inaccessible quantities such as mean curvature and Gaussian curvature as a function of time. In a next step, we develop an analytical hydrodynamic model that predicts the flows inside the oocyte as a result of the observed SCW. The mathematical model is based on the analytical solution of the Stokes equation and has the advantage that even videos with several thousand frames can now be easily analysed. Additionally, different contributions to the flows can be separated and compared to each other. We find excellent agreement between our predictions and the PIV-results. From this we can conclude that during the time of the SCW, the cytoplasm behaves as a simple Newtonian fluid on the scale of several micrometers. The model is general in the sense that it can be applied to any cell that is approximately spherical, as it is the case for the oocytes of most echinoderms and vertebrates, including mammals. Finally we use our finding to address the proposed biological function of cytoplasmic flows in starfish oocytes. We calculate the intracellular pressure field and find that it is not sufficient to account for polar body extrusion, in contrast to earlier suggestions. This is confirmed experimentally by blebbistatin experiments, which strongly reduce cell shape changes and hydrodynamic flow, but not polar body extrusion. By analysing the cytoplasmic flows we can furthermore assess the role of cytoplasmic flows for mixing of cytoplasmic components, including determinants that pattern embryonic development.

## Results

### 3D segmentation suggests rotational symmetry of SCW

**Fig 1:**
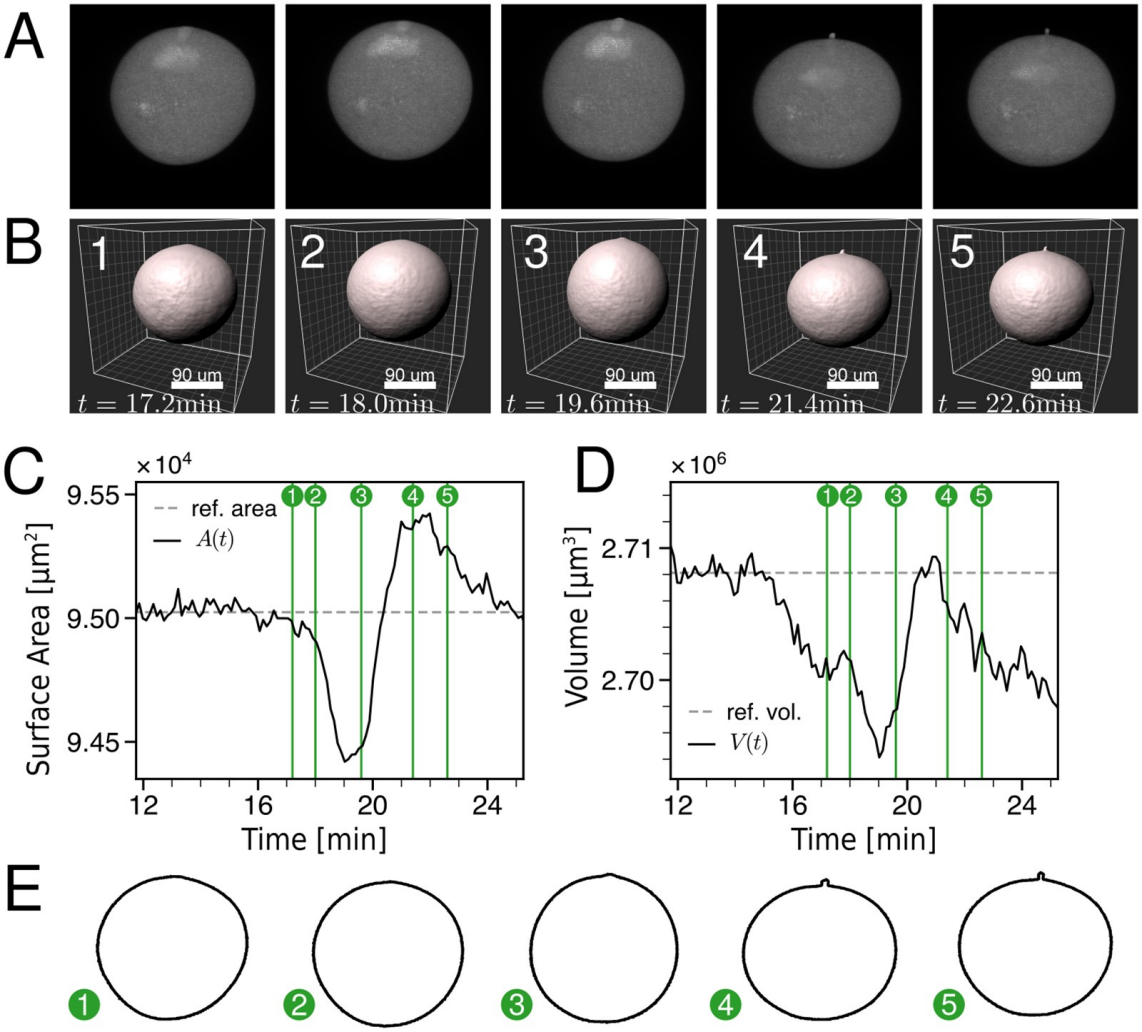
3D reconstruction of oocyte during the SCW. A: Maximum intensity projections of an oocyte during the SCW. The oocytes were filled with fluorescently labelled dextran to mark the cell volume. A video is given as S1 Video. B: The surface reconstruction shows rotational symmetry of the SCW. A video is given as S2 Video. C,D: Surface area and volume extracted from the segmentation shown in B. Both show a slight decrease during the SCW. E: Slices of the cell surface containing the AV-axis and the polar body show the typical shape changes during the SCW.

In order to image cell shape changes in 3D during the SCW, we microinjected a bright fluorescent dextran into starfish oocytes to mark the cell volume. We then used a Zeiss AiryscanFast microscope on freely floating cells to acquire one 3D image stack every 7 s over a 15 min time course including the SCW. Fig 1A and 1B show sequences of snapshots from a representative cell (full videos provided as S1 and S2 Videos respectively). While Fig 1A shows the maximal intensity projections performed with the open source image processing package Fiji, Fig 1B is a rendering of the segmentation performed with the commercial image processing software Imaris (for details, see the Methods section). In general, the cell is always close to spherical due to the large cortical tension of around 2 mN m^−1^ [21]. Close inspection shows that it takes the SCW roughly 7 min to run from the vegetal (V) to the animal (A) pole, and that the polar body starts to appear roughly 3-4 min after the wave has started. The wave itself is visible as a band of deformation running from the V- to the A-pole, confirming previous observations [21]. In Fig 1C and 1D we show the global quantities surface area and volume that can be extracted from the segmentation shown in Fig 1B. One sees that both are slightly reduced during the SCW, but then recover to pre-wave values. Interestingly, the area then shows an overshoot, which partially corresponds to the formation of the polar body, presumably because exocytosis and membrane flattening generate additional membrane area. Exocytosis of fluorescent dextran might also explain why the values for surface area and volume drop after the SCW. In Fig 1E we show the boundary contours from Fig 1A. While the shape changes are relatively small, it is apparent that they are symmetric around the AV-axis. In order to address the relation to hydrodynamic flow, we therefore turned to transmission light microscopy in 2D slices containing the AV-axis, that allowed simultaneous imaging of cell shape changes and cytoplasmic flows.

### 3D geometrical quantification of the SCW from 2D slices

**Fig 2:**
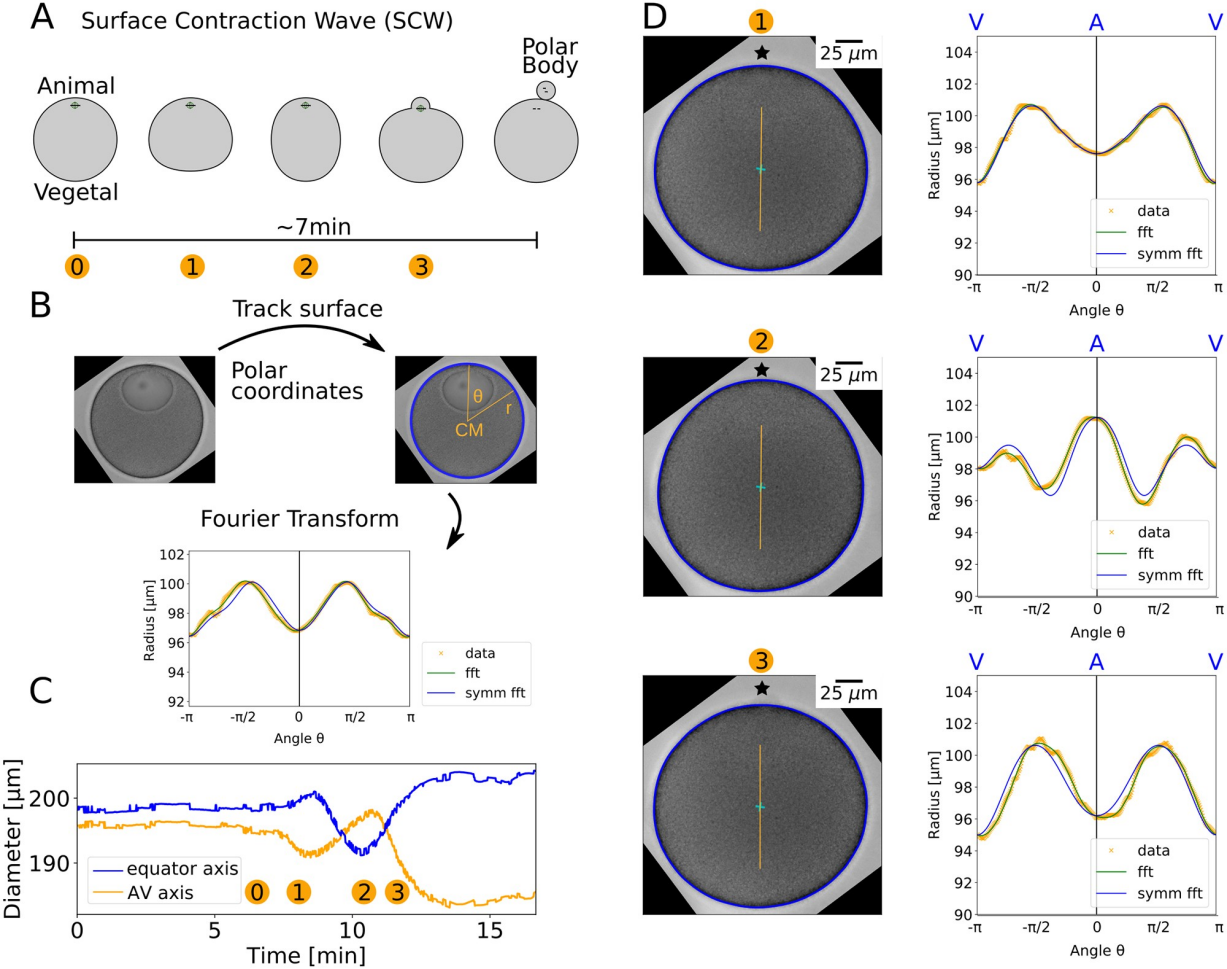
Quantification of SCW in 2D. A: We consider 2D slices through the cell volume containing the AV-axis. The SCW takes about 7 min to run over the surface. Different intermediate stages during the SCW are indicated by numbers. B: The cell contour (blue rim) was tracked with image analysis methods from transmission light microscopy images. It was then described in polar coordinates with the contour centre of mass as coordinate origin. To smooth the curve, higher Fourier modes were discarded (fft). The symmetry of the radius function was used to determine the orientation of the AV-axis and the signal was symmetrised around it (symm fft). C: The diameter of the oocyte at the AV-axis develops from a minimum via a maximum to a minimum, whereas the diameter on the perpendicular axis towards the equator shows the opposite behaviour, as expected from A. D: The radius function was defined with respect to the centre of mass (cyan cross). The AV-axis is marked as an orange line and the animal pole by a star. The surface of the oocyte shows high symmetry during different stages of the SCW. The difference between the smoothed radius function (green line, fft) and its symmetrised version (blue line, symm fft) is very small. In stages 1 and 4 there are local minima at the animal pole whereas in stage 2 we found a local maximum. A video of the SCW is given as S3 Video.

We next recorded transmission light microscopy videos of oocytes in imaging chambers at the height of maximal area and selected oocytes with the AV-axis lying in this slice (N = 24). Because immature oocytes before meiosis I have their nucleus located at the A-pole, the AV-axis can be identified before the SCW starts. In agreement with Fig 1E, in 2D we always observed the stereotypic sequence of events shown schematically in Fig 2A. Naturally, the oocytes are surrounded by a jelly coat to protect them from the sea environment. By removing the jelly coat, one can observe even larger surface deformations, but here we show the results for one representative cell with the jelly layer kept, in order to reflect physiological conditions. A description of the cell surface in polar coordinates gives a radius function *r*(*θ*, *t*) (Fig 2B). We defined polar coordinates such that the polar angle *θ* = 0 lies at the A-pole and the V-pole at *θ* = *π*. After a Fast Fourier Transform (fft), a back transform with seven Fourier modes resulted in the smooth representation shown in Fig 2B. A comparison with the backtransform using even parts only (symm fft) revealed that the radius function is highly symmetric around the AV-axis (Fig 2B). The AV-axis determined by symmetry considerations coincides very well with the axis connecting the centre of mass (CM) with the centre of the nucleus. In CM frame, during the full time course we first observed a local minimum on the AV-axis that then became a maximum and finally developed back to a minimum (Fig 2C), whereas on the perpendicular axis towards the equator the opposite behaviour can be observed. The maximal radius changes are about 10%. The high symmetry around the AV-axis apparent in Fig 2B was in fact found for the whole time course of the SCW (Fig 2D and S3 Video). Fig S1 Fig shows the surface tracking results for the complete data set (N = 24) for the time point after the SCW, when maximal shape changes were observed. Considering the full data set demonstrates that the cell shown in Fig 2 is indeed representative and that the described shape changes are very stereotypical in starfish oocytes.

**Fig 3:**
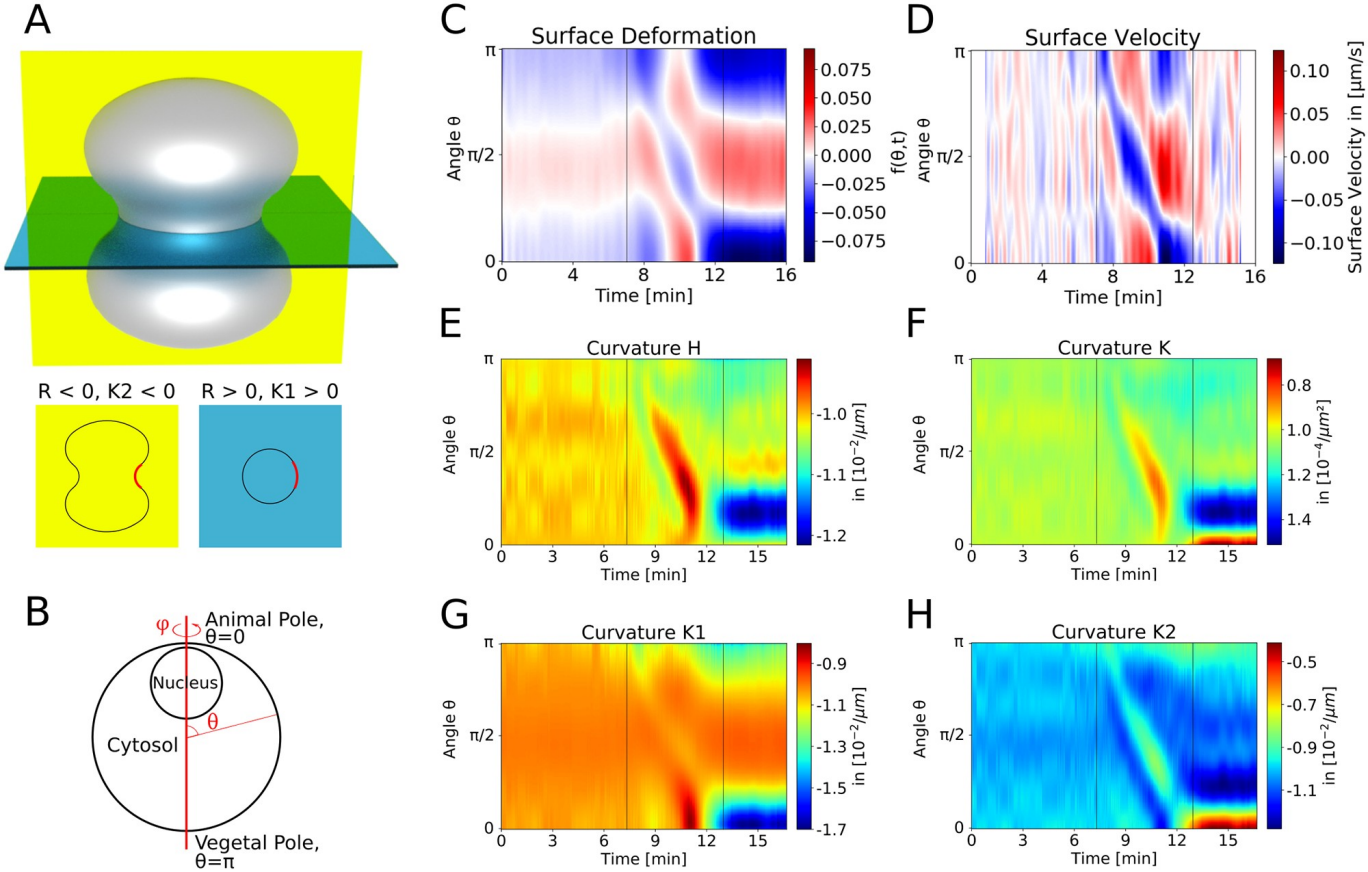
Dynamics of surface displacements and curvatures during the SCW. A: Cartoon of a cell with invaginated shape. For each direction at each point on the surface, a curvature (the inverse of the local curvature radius) can be defined. Here the two principal curvatures of the rotationally symmetric surface at the equator are shown as K2 in the yellow plane and K1 in the blue plane. At the same point they have opposite signs (red section). B: The starfish oocyte is assumed to be rotationally symmetric around the AV-axis. This makes it possible to calculate the three-dimensional shape from two-dimensional images. Thus we have access to the full set of curvatures. C,D: The normalised deformation from a perfect spherical shape (C) and the radial surface velocity (D) clearly define the SCW-speed. E-H: The kymographs of mean curvature (H), Gaussian curvature (K) and the two curvatures in *φ*-direction (K1) and in *θ*-direction (K2) confirm that the SCW can be described as a band of local flattening running over the oocyte. Evaluation of the Gaussian curvature K demonstrates that the surface transiently evolves towards a saddle shape.

As suggested by Fig 1 for the 3D data and by the symmetry in the 2D slices containing the AV-axis shown in Fig 2, we assume rotational symmetry of the oocyte around the AV-axis to quantify the geometrical changes during the SCW on the basis of the 2D slices. Fig 3A shows a cartoon of the invaginated shape expected for a strong SCW passing the equator. Using differential geometry, one now can calculate the 3D curvature of the cell surface from the 2D data (for details compare Methods section). For example, at the equator of an invaginated shape the two principal curvatures should have opposite signs. In the following we calculate all local geometrical properties of the 3D surface from the 2D data as a function of the polar angle *θ* (Fig 3B). Fig 3C shows a kymograph of the normalised difference between the oocyte’s shape and a perfect sphere. We describe the surface by a radial coordinate *r* = *a*(1 + *f*), where *a* is the radius of a reference sphere and *f* describes the deviation from the spherical reference case. One sees that the beginning and end of the wave are clearly visible at times around 7 and 13 min, and that the wave velocity (slope) is well defined. Fig 3D shows the kymograph of the radial surface velocity in centre of mass frame of reference, which shows the same pattern. The deformation persists for several minutes after the SCW has passed (Fig 3C and 3D, S3 Video). The mean curvature H and the Gaussian curvature K (Fig 3E and 3F, respectively) show similar patterns as do the deformation and velocity kymographs. Both curvatures change towards zero because as the wave passes by, the invaginated part at the equator acquires a saddle-shape (a perfect saddle has H = 0 and negative K). The reduced curvatures show that the SCW can be described as a band of local flattening running from V-pole to A-pole, as concluded earlier from a purely two-dimensional analysis [21]. K1 and K2 shown in Fig 3G and 3H show the principal curvatures in *φ*-direction and *θ*-direction, respectively (blue and yellow plane in Fig 3A). The wave is most pronounced in K2, which corresponds to the curvature directly visible in the imaging plane (Fig 3H). Fig S2 Fig uses the example of K2 to show that the 3D data presented in Fig 1 gives similar results when subjected to the procedures described here for the 2D data. However, the resolution and throughput is much better in the 2D case.

### Flow and pressure fields can be predicted from surface movement

**Fig 4:**
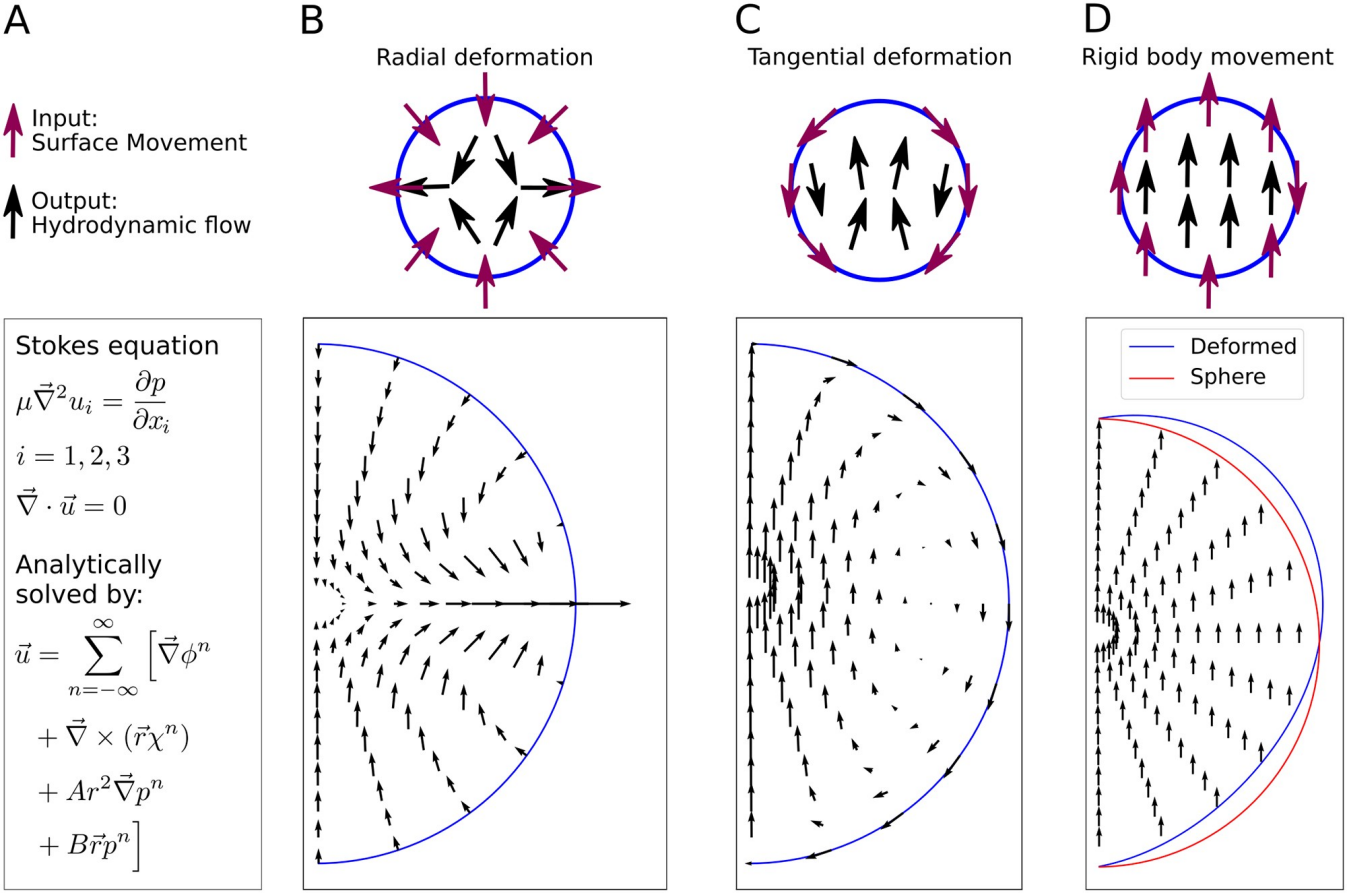
An analytical hydrodynamic calculation predicts flows from surface displacements. A: The flows inside the oocyte are modelled as Stokes flows of an incompressible Newtonian fluid. We have constructed an analytical solution for the flows inside a moving sphere-like boundary. We applied it to the flows inside a rotationally symmetric object. The problem reduces to the calculation of three fields *ϕ*, *χ* and *p* from the surface movement as given in the Methods section. B: The model predicts the flows for arbitrary radial surface movement, exemplified here by a surface movement where there is influx at the poles and outflux at the equator. C: Arbitrary tangential surface movement can be included, exemplified by constant surface movement from one pole to another. D: Due to linearity of the Stokes equation different contributions to the flows can be separately calculated and added up so that rigid body movement can be predicted from adding up radial and tangential flows. The model is extended using perturbation theory to also deal with non-spherical shapes. This is exemplified by rigid body movement of a deformed sphere (blue). An undeformed sphere is shown as reference (red).

After the geometric description of the SCW, we next asked how the cell shape changes relate to cytoplasmic flows. We therefore implemented an analytical model for the hydrodynamic flows inside a spherical shell and applied it to the problem of flows inside the deforming oocyte. With this model we are able to predict the flows of a Newtonian, incompressible fluid at low Reynolds number. The Stokes equation was solved analytically with no-slip boundary conditions on the boundaries extracted from the experiments (Fig 4A). We note that no-slip boundary conditions are commonly applied when solving the Stokes equation of hydrodynamic flows in soft matter and biological systems [29–31]. Potential slip lengths at the boundary are expected to be in the nanometre range and therefore can be neglected in our context. Although volume and surface area are known to change during the time course of the SCW, this does not matter for our computational procedures because the Stokes equation does not have memory and can be solved anew for each time point.

In principle the model can predict flows even if there is no rotational symmetry in the problem. Three examples are given in Fig 4B–4D, where rotational symmetry is assumed for all three setups. The effects of arbitrary tangential and radial surface movement can be calculated separately as shown here for a radial movement (Fig 4B) and a tangential movement (Fig 4C). Due to linearity of the Stokes equation, different contributions to the flows can be added as it was done for rigid body movement in Fig 4D. In this case, radial and tangential surface movements were added to obtain the constant flow field for rigid body movement. Additionally, the model is able to predict the flow field inside a slightly deformed sphere by a perturbation ansatz as done for Fig 4D. Together with the flow field, this model gives the pressure field (*p* from Fig 4A), that is developing inside the fluid. In the Methods section we give analytical expressions for these quantities, and additional information is provided in the S1 Appendix.

**Fig 5:**
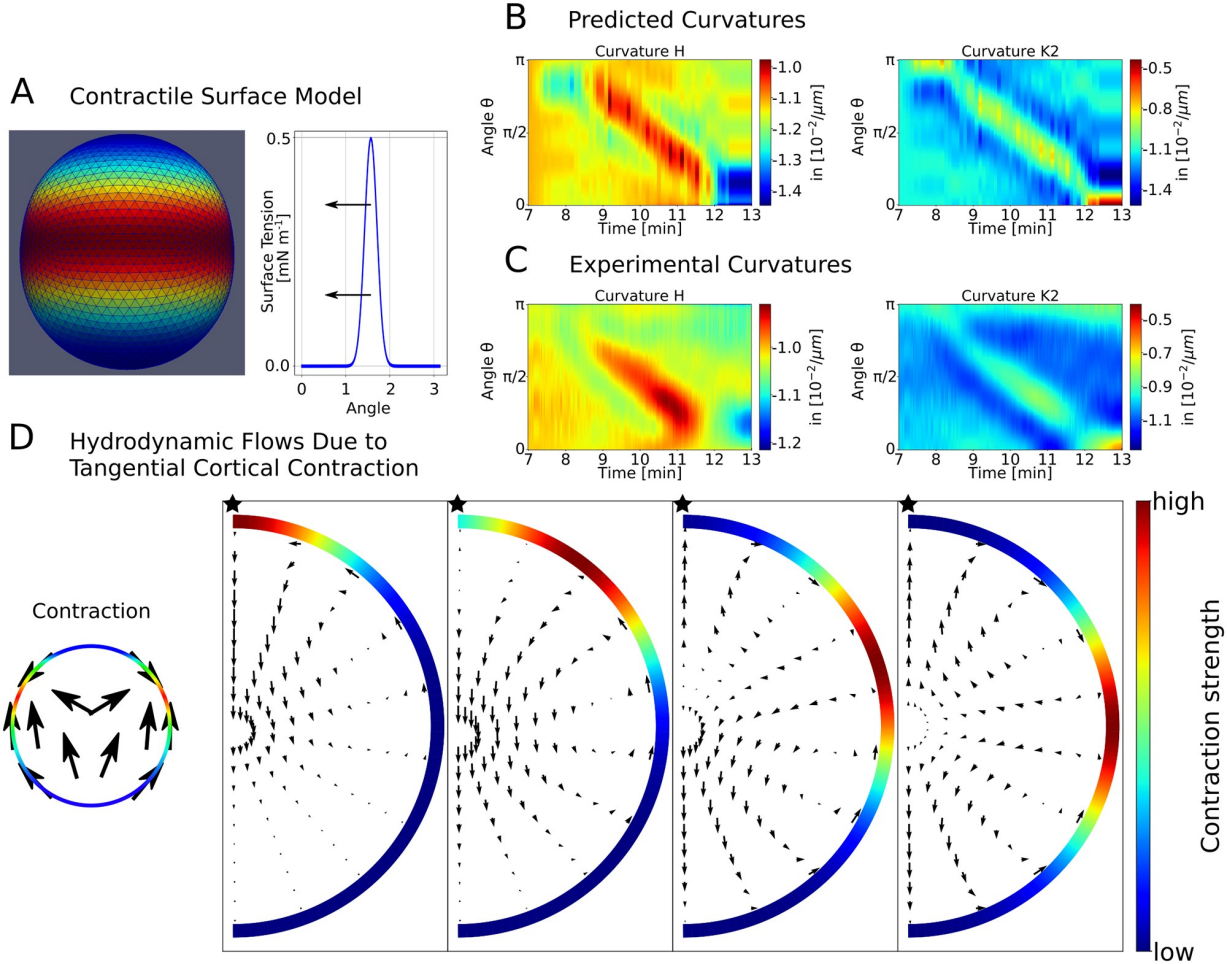
A contractile surface model predicts shape changes during the SCW. A: For each time step a surface Hamiltonian for a spherical shape with varying surface tension is minimised. The surface tension follows a Gaussian profile that is moved over the surface with changing strength. The predictions of the model in B show similar features as a zoom-in to the experimental curvatures during the SCW in C. D: The contractile model can also predict tangential flows in the cortex, that in turn can couple to the hydrodynamic flow, in addition to flows caused by the radial deformations as explained in Fig 4. The tangential surface movement due to the Gaussian-shaped local contraction of a viscous medium is analytically calculated. The tangential surface movement is used as an input for the hydrodynamic model. The effect of different contraction positions on the flow pattern is small. This can be seen from the flows (arrows) due to a rotationally symmetric surface contraction (red colours) around the AV-axis (star).

In order to address the mechanical basis of the surface deformations, we used a contractile surface model that describes a band of increased surface tension moving over the oocyte cortex (Fig 5A), following [21] and as confirmed now in 3D by Figs 1 and 3. For each time step the shape of the surface is computed numerically by minimising the appropriate surface Hamiltonian. In our specific case, we used a Gaussian-shaped contraction band travelling with constant angular speed and constant width but changing amplitude (see Fig S3 Fig). We adjusted the strength and width of the contraction band to visually reproduce the curvatures found in experiment (Fig 5B and 5C). The angular width of the contraction band was determined to be 72°. Our contraction model also allowed us to predict the tangential surface movement. Here we used the model of a flowing viscous surface as previously applied to the cortex of *C. elegans* embryos [32]. The dynamics of the SCW are sufficiently slow (timescale of minutes) so that short time elastic contributions can be neglected. We combined the analytical solution for the tangential surface movement with the radial movement of the surface obtained before from modelling the shape changes. We then used the resulting tangential surface movement as an input for the hydrodynamic model in order to predict the internal flows that should result from a localised Gaussian contraction band. For four different positions these are shown in Fig 5D. These flows are rather robust to variation of parameters, such as width of the contraction band and the spatial decay length of the velocity. More details are given in the Methods section.

### Cytoplasmic flow is a direct consequence of surface movement

**Fig 6:**
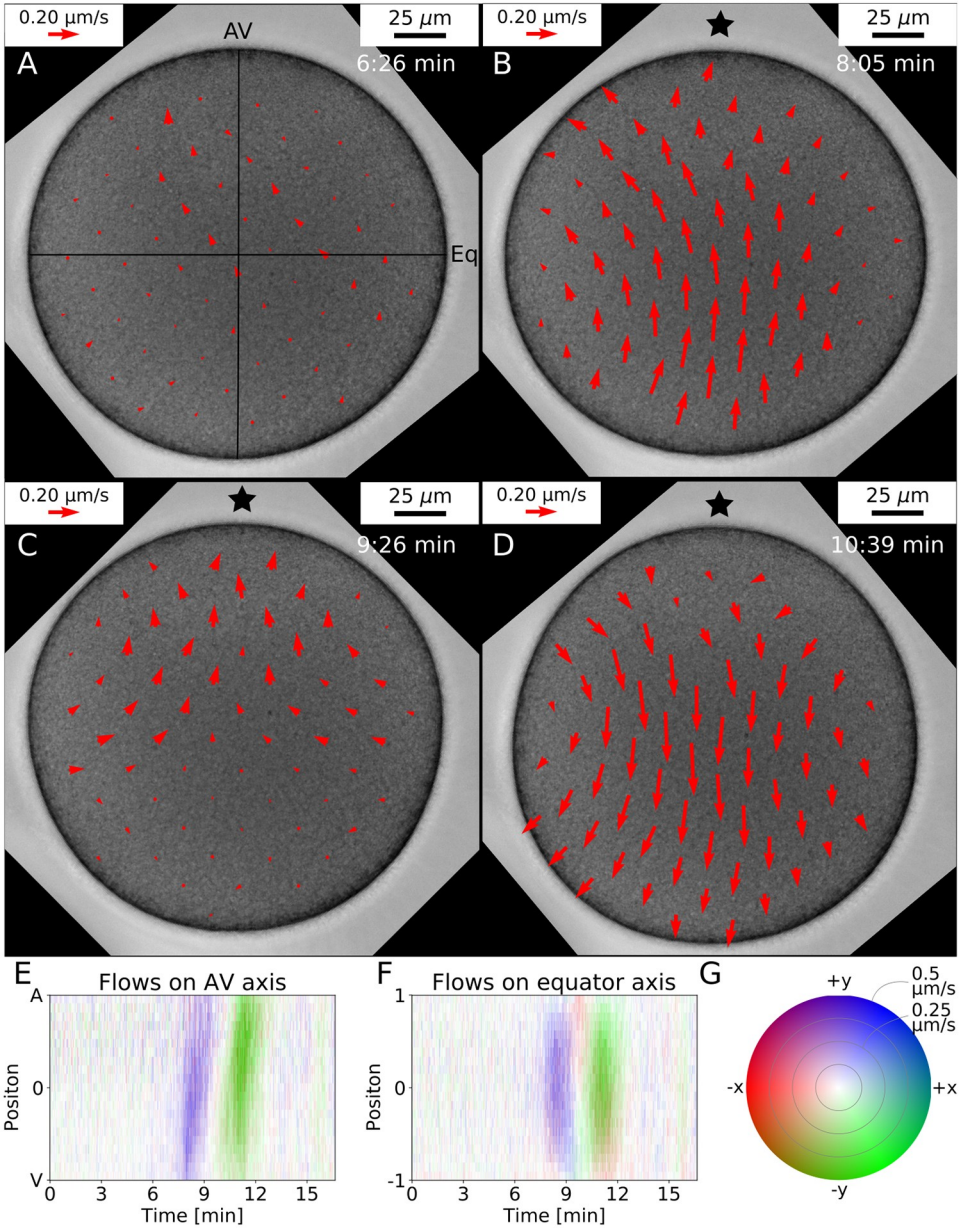
Experimentally measured cytoplasmic flows. The flows inside the starfish oocyte can be measured using a standard PIV-algorithm. A: Only small irregular flow patterns (red arrows) can be detected as long as no surface movement is present. B: The flows point mainly from the vegetal pole to the animal pole. C: The flows now come from the equator and point in direction of both poles. D: The flow goes back towards the vegetal pole. A video of the flows for the full SCW is given in S4 Video. E-G: Quantification of the flows on the AV-axis (E) and on the perpendicular axis (F, ±1 correspond to equator right and left). The colour intensity encodes the flow velocity while the colour tone indicates the flow direction as explained by the polar colour map in G.

We next compared our theoretical predictions with the cytoplasmic flow as measured by PIV [33, 34] applied to the 2D transmission light microscopy data. Here we exploit the fact that in starfish, the naturally occurring yolk particles (1 μm to 2 μm in size) follow the hydrodynamic flow and produce the contrast needed for the PIV-algorithm. Using this method, internal flows were quantified reliably during the full SCW with a spatial resolution of 10 μm (Fig 6, S4 Video, see Methods section for the PIV details). We find that during the SCW the flows at first drive the cytoplasm from vegetal to animal pole, then the flows reverse and drive the fluid back to the vegetal pole. The flows have curved streamlines and visually show a high degree of symmetry around the AV-axis (Fig 6A–6D). Also the flows show a clear wave-like behaviour which is further confirmed by the kymographs in Fig 6E and 6F for the flows along the AV-axis and on the axis perpendicular to it, respectively (colour code given as Fig 6G).

**Fig 7:**
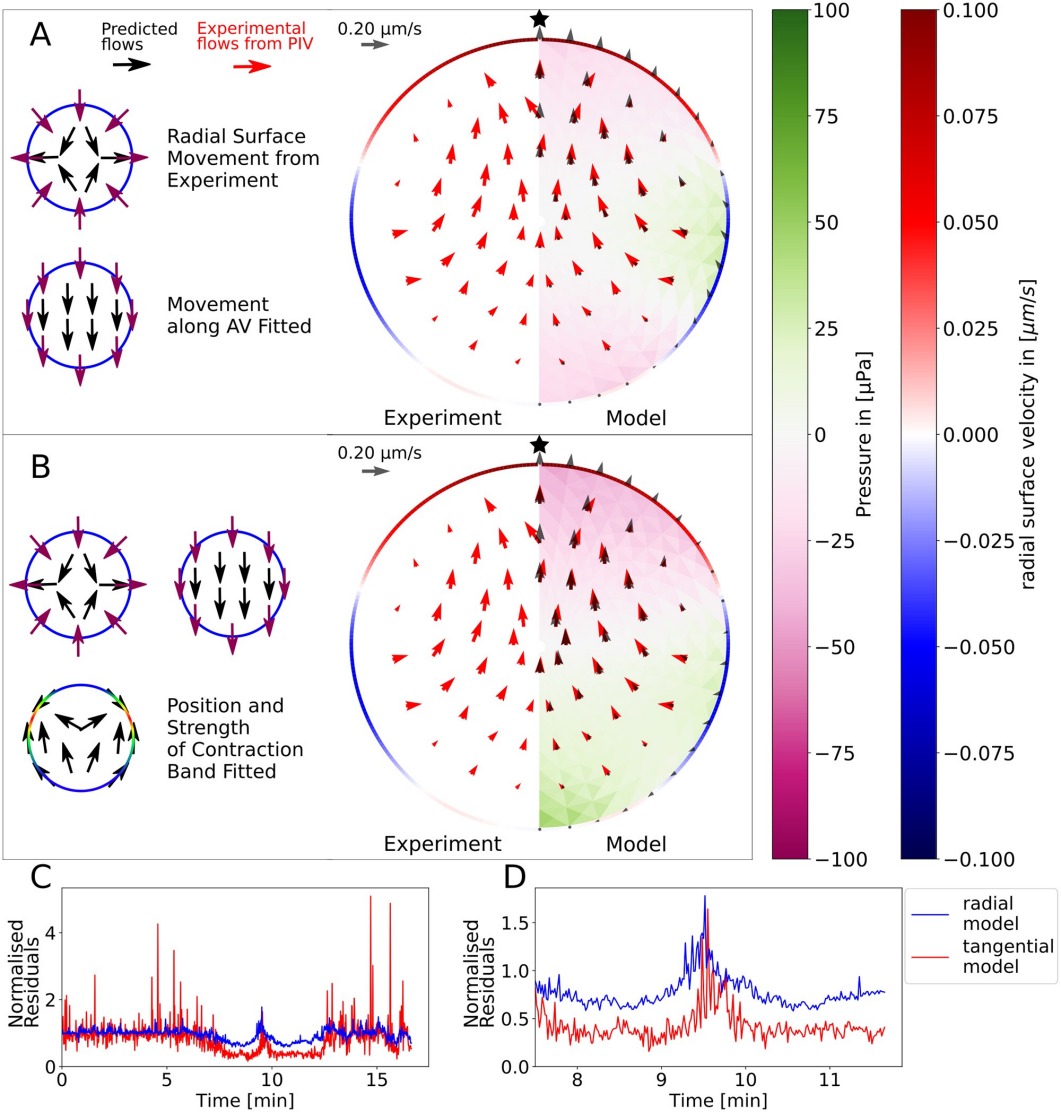
Comparison of the hydrodynamic model with experimental flows. A: The experimentally measured flows (red arrows) are compared with flows predicted by the hydrodynamic model (black arrows). The hydrodynamic model also predicts the internal pressure field (purple to green colours) that is experimentally not accessible. The model is based only on the radial surface movement obtained from experiment (red to blue rim). Whole body movement parallel to the AV-axis is fitted. B: The model’s accuracy can be improved by taking into account tangential surface movement as an effect of local surface contraction. Now the position and the strength of the contraction band are fitted for each time step individually. Videos of the SCW predicted by the radial and tangential displacement models, respectively, are given as S5 and S8 Videos. C,D: The quality of the tangential displacement model quantified by the normalized residuals is significantly better during the wave (C whole time course, D zoom in to wave).

With the hydrodynamic model introduced above the internal flows during the SCW can be predicted from the surface movement of the oocyte and compared with the measured flows. In the radial displacement model the radial surface movement obtained from experiment is used as sole input. This surface movement thus sets the boundary conditions for the solution of the Stokes equation for the cell interior. In Fig 7A a comparison between the flows predicted by this model and the experimental flows is shown. The radial surface movement is obtained by comparing the contour points of subsequent frames. We fitted the movement of the whole cell along the AV-axis to the experimental flows as the radial surface movement was calculated in the cell centre of mass frame. This model is able to explain the strength and the general shape of the flows during the SCW (Fig 7A). The velocity of the internal flows is of comparable size to the radial surface velocity. The good correspondence between the predicted and the experimental flows justifies the assumption of modelling the cytoplasm as an incompressible Newtonian fluid on the 10 μm scale. In addition to the flows, the model predicts the internal pressure field which scales linearly with the fluid viscosity. The viscosity of the cytoplasm was determined to be about 5mPa s by comparing the diffusion coefficient of fluorescently labelled dextran in water and in the cytoplasm using fluorescence correlation spectroscopy (FCS, compare Methods section). The pressure difference inside the oocyte is on the order of 200 μPa. A full video comparing experiment and this model can be found as S5 Video.

Visual inspection of Fig 7A and S5 Video shows that the intracellular flow might have a stronger tangential component close to the cortical surface than predicted by the radial displacement model. Indeed experimentally often a strong tangential flow component can be observed (S6 and S7 Videos). However, it is very difficult to quantitatively measure tangential surface movement experimentally. We therefore used the mechanical contraction model to predict possible tangential flows (compare Fig 5). Adding the effect of tangential surface movement to the predictions of the radial displacement model resulted in the tangential displacement model. We next fitted the strength and the position of a Gaussian contraction band to the experimental flows (see the Methods section for further details). The result for one time step is shown in Fig 7B where the quality of the modelled flows further improves by taking tangential surface movement into account (full comparison given as S8 Video). In Fig S4 Fig a detailed view for additional time steps for this method are shown. The maximum internal pressure difference inside the cell is again found to be around 200 μPa, but now the gradient is more clearly directed along the AV-axis. The addition of the tangential movement to the model roughly doubled the accuracy of the model during the SCW, as shown in Fig 7C and 7D. Here the plotted normalised residuals are defined as the root of the squared difference between modelled and measured flows normalised by the strength of the measured flows. The increase in agreement becomes apparent during the wave (full time course in C, zoom in to the wave in D), when significant flows are measured. Before and after the wave, the normalised residuals fluctuate around unity by definition.

In summary, we conclude that the flow pattern during the SCW in the cytoplasm is driven purely by the surface movement. This finding implies that the cytoplasm behaves like a Newtonian fluid during this stage of the development. This is surprising given the microscopic complexity of the cytoplasm and reflects that during SCWs, the cytoplasm of the starfish oocytes does not contain any relevant (cytoskeletal) structures on the scale of several micrometers. We also note that the model predicts a significant tangential component, which experimentally cannot be measured easily.

### The measured flows do neither lead to mixing nor to polar body extrusion

**Fig 8:**
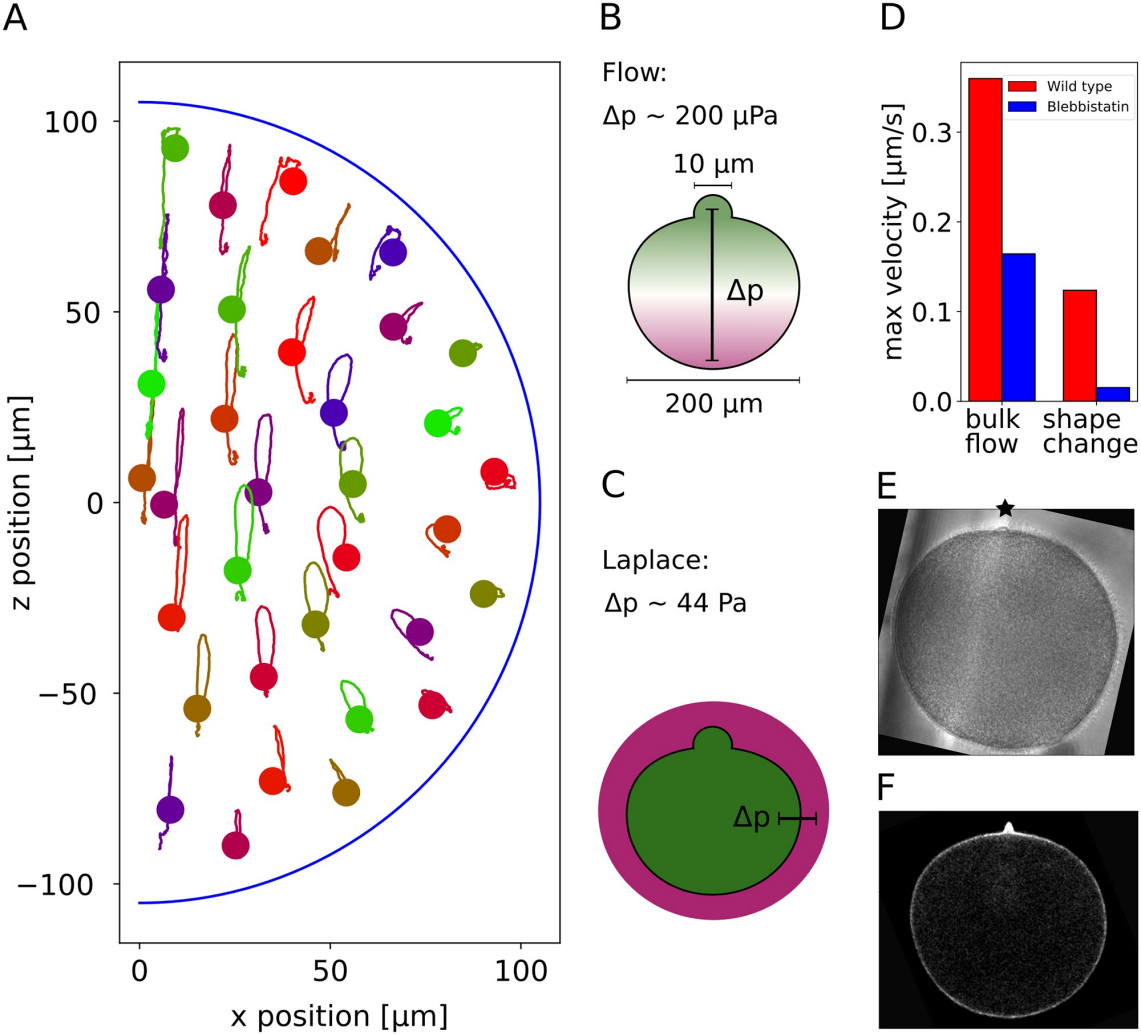
Mixing hypothesis and polar body formation. A: A particle starting at the dot follows the shown line during the SCW. Most trajectories end approximately at their starting point and follow similar paths as their neighbouring ones. Therefore mixing of the cytoplasm can be excluded as a function of the SCW. B,C: It has been suggested that the polar body is pushed out by the SCW due to build up of high internal pressure. Our hydrodynamic model shows that the SCW leads to a locally higher pressure at the animal pole of several hundred μPa. If the polar body was pushed out by a blebbing mechanism, the pressure difference between inside and outside would need to be of the order of several hundred Pa at the animal pole. Therefore the pressure due to the SCW can be excluded as a mechanism for polar body extrusion. D: Both the deformation speed and the internal flows of a blebbistatin-treated cell are much weaker than in the wild type cell. E: A polar body is formed even with blebbistatin-treatment. Full videos are given as S9 and S10 Videos. F: A potential mechanism for polar body extrusion is local actin polymerisation. The actin of the oocyte was labelled with utrophin which makes a local accumulation of actin visible at the position of the polar body (S11 Video).

We finally discuss the possible biological functions of the observed phenomena. Mixing of the cytoplasm has been proposed as a potential biological function of flows in various species [15, 16, 32]. To elucidate the role of the SCWs for intracellular mixing, we calculated trajectories of fictitious particles embedded into the induced flow fields. We observe that most simulated tracer particles return to their original position after the SCW (Fig 8A). Only regions near the animal pole show significant displacement due to the fact that the cell is still highly deformed at the end of imaging as shown in the kymographs in Fig 3. Therefore intracellular transport of particles on the scale of several micrometers can be ruled out as a potential function of the SCW. Additionally we do not observe any mixing in the trajectories due to the SCW as the streamlines have the same shape and move in parallel to each other.

Having access to the flow field and the pressure field, we are also able to revisit the earlier suggestion that that the SCW drives extrusion of the polar body [4]. If the polar body was pushed out by intracellular pressure, as in blebbing [35, 36], large pressure differences between inside and outside would be required to be built up by the hydrodynamic flow. Before the SCW, the pressure difference between inside and outside of the oocyte can be estimated from the Laplace law, Δ*p* = 2*γ*/*R*. The tension in the oocyte surface has been measured by micropipette suction experiments to be around 2 mNm^−1^ [21]. With a cell radius of *R* = 90 μm, this gives a pressure difference of 44 Pa. This value is similar to but somehow smaller than typical values for human cells, which have been reported to be of the order to several hundred Pa [35, 37], because they have smaller sizes but comparable levels of cortical tension. If hydrodynamic flow now led to polar body extrusion, one would expect at least a contribution of the same order of magnitude as 44 Pa. The internal pressure difference predicted by our hydrodynamic model however is only about 200 μPa, which is five orders of magnitude smaller. Therefore we conclude that the internal pressure difference due to the SCW is negligible compared to the total pressure difference needed for polar body extrusion. This is illustrated in Fig 8B and 8C. Bischof *et al*. showed that the SCW is significantly weakened when non-muscle myosin II contractility is inhibited by blebbistatin [21]. In agreement with our conclusion that cell shape changes drive hydrodynamic flow, we find experimentally that under blebbistatin, also hydrodynamic flows are weakened (Fig 8D and S9 and S10 Videos). Yet these cells do form a polar body (Fig 8E), although they are not able to pinch in the neck. By imaging fluorescently labelled actin, a clear accumulation of actin at the position where the polar body is formed becomes visible (Fig 8F and S11 Video). This suggests that actin polymerisation is the underlying mechanism to generate polar body formation, similar to the mechanism of polar body extrusion in *Xenopus* [38].

## Discussion

SCWs have been described in various species, including axolotl, frog and starfish, for more than 30 years [3]. Recently Bischof *et al*. have revealed the underlying molecular mechanism for starfish [21], explaining the travelling band of local flattening by local activation of the Rho-module coupled to the cell cycle by a cdk1-gradient. However, this analysis did not consider the consequences for the cytoplasmic flow. Here we provided a full quantitative analysis of the hydrodynamics during the SCW and therefore for the first time arrive at a complete description of the SCW during starfish meiosis.

We first provided a full 3D analysis of the cell shape changes during the SCW. Imaging oocytes labelled with fluorescent dextran revealed that they are roughly axisymmetric around the AV-axis, thus justifying a 2D approach in which we only image 2D slices containing the AV-axis with transmission light microscopy, which allowed us to simultaneously measure cell shape and hydrodynamic flow. The assumption of rotational symmetry around the AV-axis has been suggested before [25] and is also supported by the observation that the orientation of the SCW is fully determined by the position of the nucleus [21]. Here it is further supported by the observations that both cell shape changes and hydrodynamic flow are highly symmetric in the plane containing the AV-axis. Assuming axisymmetry and using differential geometry, we are able to calculate 3D curvatures from 2D data. Our quantification of cell shape changes in all directions confirms the results of Bischof *et al*. [21], and in addition gives also mean and Gaussian curvature, which are important to fully evaluate the relevant surface energies [39]. By using the different curvatures, we also were able to estimate the angular width of the contraction band to be about 72°.

In principle, one also can calculate surface area and volume for the rotational symmetric surface assumed here (compare Methods section). For the cell shown in Fig 3, we found that surface area and volume reduce by about 3% and 4%, respectively, during the SCW. Regarding our whole data set (N = 24, including the cases with the jelly removed), we found reductions by (8 ± 2)% and (12 ± 3)%, respectively (Fig S5 Fig). Although the reduction during SCW and the following overshoot in area are in agreement with the 3D data from Fig 1, the large magnitude of these changes and the overshoot in volume (which here is calculated from the same radius data as the area) are probably artefacts of the 2D method, where oocytes are confined in imaging chambers. However, this does not directly affect the main focus of our work, namely analysis of the hydrodynamic flow, for which surface area and volume are not essential. In the future, one might envision a more general modelling framework that also includes water flow through the cell surface. This however requires more precise measurements of surface area and volume, and a corresponding image processing pipeline, that was not the focus of the work reported here. In general, we anticipate that very good resolution was required to resolve these effects. If one assumes an outflow of Δ*V* = 10^4^ μm^3^ in *T* = 2 min and a surface area of *A* = 10^5^ μm^2^ (compare Fig 1), then the resulting movement of the surface would be *v* = Δ*V*/(*AT*) = 10^−3^ μm/s, much smaller than the typical speed of surface movement around 0.2 μm/s. With the current resolution, this effect can thus be safely neglected.

The main focus of this work is the hydrodynamic flow during SCWs. Hamaguchi and Hiramoto have given a pictorial description of the flows during the SCW in their seminal paper in 1978 [4]. Since then, methods to quantitatively measure flow have evolved tremendously. We measured the internal flows by PIV on transmission light microscopy recording, which gave us a quantitative description of the flows with high temporal and spatial resolution. We used the naturally occurring yolk particles as tracers of the internal flows which circumvents the use of artificial tracer particles. We found that the SCW and the flows resulting from it can both be described as direct physical consequences of a band of local contraction travelling from vegetal to animal pole (S1 and S2 Videos). Our work not only provides a quantitative description of the internal hydrodynamics within the oocyte at high resolution, but also explains the origin of the induced flows.

The model is based on an analytical solution of the Stokes equation in spherical geometry. The assumption of incompressibility and low Reynolds number (Re ≈ 2 × 10^−4^) seems to be satisfied for the starfish cytoplasm. Additionally the cytoplasm is described to behave as a Newtonian fluid. The good agreement between measurement and the model shows that the cytoplasm behaves as a simple fluid regardless of its complex structure. Such behaviour is also known for *Drosophila* [14]. Even though the volume of the cell changes over time (Fig 1), the assumption of incompressibility can be made as explained in the Methods section. The only input of the model is the oocyte shape and its surface velocities which serve as boundary conditions. We assumed no-slip boundary conditions in order to couple the internal flows with the surface movement. In addition to the internal flows the model predicts the pressure field inside the oocyte. In a simple version our model has only one free parameter, which is the movement of the centre of mass along the AV-axis. This radial displacement model already explains most features of the flows. By extending the model to tangential flows due to local contraction of the cell surface, we could show that tangential surface flows towards the contraction band should also play a role for the internal flows. In the future, this prediction has to be confirmed experimentally by imaging actomyosin flow in the plane of the cortex, as has been done earlier for *C. elegans* [32]. In contrast to the case of *C. elegans*, however, here the spatial actomyosin distribution varies strongly in time (as shown explicitly in S12 Video) [21]. Similar dynamical distributions might be at work in other organisms and our model with both radial and tangential displacements should be applicable to any approximately spherical cell.

Cytoplasmic flows have been shown to be important in the development of multiple organisms [17, 32]. Recently Boquet-Pujades *et al*. have introduced a method to calculate an internal pressure field by numerically solving Stokes equation [27]. The great advantage of our analytical model is the feasibility to compute the flow field for videos with several thousand frames, which is computationally very demanding for a numerical procedure. Additionally, different contributions to the flows can be studied individually using our method. For instance, we are able to directly discriminate the effects of radial flows from tangential flows. We note that our model can be used in the future to predict experimentally inaccessible internal flows for non-transparent cells, as it is the case of the *Xenopus* oocyte, by tracking its surface movement. This is especially interesting because the cytoplasm of *Xenopus* seems to be characterised by a spatially highly structured distribution of determinants.

Our hydrodynamic model can explain the shape and strength of the internal flow field during the SCW. This clearly shows that the flows are a physical consequence of the surface movement. The surface movement is explained by the model resulting from a band of locally increased surface tension moving over a contractile surface [21]. It is known that the cytoplasm of oocytes can behave as an active fluid [40]. However, these contributions to the flows are much smaller compared to the flows driven by the SCW and that we are able to predict with our hydrodynamic model. This shows that during the SCW internal cytoskeletal forces are smaller than the forces due to the surface movement. This is in contrast to findings in other species such as *Drosophila* [14] and *C. elegans* [22], where a complex internal biochemical machinery is involved in flow generation. These cells do not have the possibility to undergo large surface deformations as they are surrounded by a stiff egg shell, in contrast to oocytes of starfish, Amphibia, fish, mouse and other mammals. Indeed, the shape of the oocytes is determined by the cortex in these species of the deuterostome group of animals. Therefore cortical deformations can lead to strong internal flows in oocytes of these species.

Hamaguchi and Hiramoto explained polar body extrusion as the result of a locally increased pressure produced by the SCW [4]. By quantifying the pressure field inside the oocyte we demonstrated that it is five orders of magnitude smaller than the pressure difference between inside and outside of the oocyte. Importantly, we have used a value of 5mPa s for the viscosity that we have measured directly with FCS. The fact that this value is close to the one of an aqueous solution again suggests that no relevant cytoskeletal structures are present in the cytoplasm during the stage of the SCW. We conclude that the pressure gradient generated by hydrodynamic flow is too weak as to explain polar body extrusion. We tested this conclusion experimentally by myosin II inhibition, which reduced both shape changes and hydrodynamic flow, but did not abolish polar body extrusion. This result agrees with previous studies based on mechanical manipulation [23] and results known from other species [41, 42]. We further showed that actin polymerisation is the most likely mechanism for polar body formation. The main function of the SCW then might be coordination of the pinching off of the polar body, which depends on actomyosin contractility.

Mixing or moving substances inside the cytoplasm is a common biological function of cytoplasmic flows [16, 17, 32]. This becomes especially interesting for systems at low Reynolds number. In this regime hydrodynamic flows driven by movement obeying time reversal symmetry cannot lead to any mixing. Thus mixing is only driven by diffusion or by internally driven flows. Diffusion is a slow process in large systems, though. The SCW of starfish in contrast clearly breaks time reversal symmetry, because it proceeds in one direction only, from the V-pole to the A-pole, and therefore cytoplasmic mixing becomes a potential function of the SCW. In particular, the kymograph from Fig 6E shows that the two periods of upflow and downflow are not simply mirror images of each other, because the downflow does not result from squeezing from the top (downward slope in the kymograph), but rather from relaxation at the bottom (upward slope in the kymograph). Here we could show by simulated particle-tracking that nevertheless after the full SCW most particles end up where they started, because the directional movement of the SCW combines with the spherical symmetry of the soft shell such that the second half of the SCW counteracts the effect of the first half. This excludes intracellular transport of particles of the size of the yolk particles as a potential function of the SCW. Significant displacement of particles can only be detected near the animal pole, where the cell remains deformed. In addition, trajectories of nearby particles are to a large extent parallel to each other. Therefore we can exclude that the SCW contributes to significant mixing of the cytoplasm. However, the wave could still lead to accumulation of constituents much smaller than the yolk particles, which would need to be tested in the future by tracking the movement of fluorescently labelled markers. It could well be that on that smaller scale, the cytoplasm does not behave like a simple Newtonian fluid any more, but like a poroelastic medium as suggested earlier for human cells [35].

Our results show that hydrodynamic flows arise each time when major cell shape changes occur, including cell division, migration and spreading. This also implies that hydrodynamic flow might be induced by artificially effecting cell shape changes, e.g. by optogenetics [43]. At the other extreme, one could induce hydrodynamic flows by purely physical means, thus decoupling them from the biochemistry related to cell shape changes [22]. Together, these new developments open up many perspectives to elucidate if hydrodynamical flows in specific systems have a biological function or are simply by-products of cell shape changes.

## Materials and methods

### Bat star

For the study of SCWs the oocytes of bat star (*Patiria miniata*) were used. They are obtained once a year from Southern California Sea Urchin Co., Marinus Scientific, South Coast Bio-Marine, or Monterey Abalone Co. and kept in sea water tanks at the EMBL marine facility. They are kept at 15 °C and fed once a week with shrimp.

### Sea water

Sea water was made by dissolving red sea salt mix in water and filtering the water. Ca-free seawater was made from 437 mM NaCl, 9 mM KCl, 22.9 mM MgCl2 · 6 H_2_O, 25.5 mM MgSO_4_ · 6 H_2_O, 2.1 mM NaHCO_3_.

### Oocyte harvesting, preparation and maturation

Oocytes are isolated from a biopsy of the ovaries that is obtained from the dorsal side of a starfish arm using a surgical biopsy puncher. The biopsy is put into calcium-free sea water at pH 8 with 50 mM L-Phenylalanine (Sigma) added for 15 min to 20 min. This prevents unwanted oocyte maturation. The biopsy is transferred to filtered sea-water with 100 μm Acetylcholine (Sigma). This leads to a contraction of the ovaries, so that the oocytes are expunged from the ovary. Oocytes are kept in plastic Petri-dishes with filtered sea water at 14 °C for up to 3 days. For most cells in the data set, the jelly coat was removed by either treatment with actinase (30 min in 0.1 mg/ml and then 3 times washing with FSW) or by Filtered Seawater adjusted to pH4 (3 min incubation and 3 washes). Maturation is induced by adding 10 μM 1-Methyladenin (Sigma).

### Inhibitor treatments and actin visualisation

To inhibit non-muscle myosin II activity, oocytes were treated with 300 μM (-)-Blebbistatin (Abcam, stock in DMSO). Cells were pre-treated for 1h before application of hormone. To visualise actin filaments, oocytes were injected with mRNA for Utrophin-CH domain-mEGFP [44] (construct gift from Bill Bement). Injections were performed as described in [45]. Injected oocytes were incubated overnight at 13°C to allow for expression of fluorescent proteins before being matured and imaged as described above.

### 3D imaging

Full 3D imaging of the oocyte was performed on a Zeiss LSM 880, using the Airyscan fast mode, 600 Hz scan speed, with a 25x water-immersion objective. For this, cells were injected with Alexa647-labelled 10kDa Dextran as a contrast agent, treated with maturation hormone and placed freely into a glass-bottom dish before imaging. Z-slices with a distance of 2 μm were taken through the whole oocyte continuously throughout meiosis.

### Surface reconstruction of 3D volumetric imaging data

The surface reconstruction was performed with the commercial microscopy image analysis software Imaris (Imaris 9.2.0, Bitplane AG, Oxford Instruments, https://bitplane.com). For this purpose the raw data was loaded into Imaris as a series of z-stacks. The pixel intensity showed a global decrease with increasing z-stack direction. To compensate this loss of signal for deeper optical sections, we applied an attenuation correction. We used a ratio of 256 to 30 from front to back (in z-direction), to linearly interpolate pixel intensities, resulting in overall homogeneous pixel intensity levels for all regions of interest containing a signal (foreground). The two values stand for the mean intensities in the front layer compared to the back layer of the full z-stack. Then we used this corrected data set to use Imaris’ surface reconstruction algorithm. This algorithm applies the marching cubes algorithm from Lorensen and Cline [46] and uses an absolute pixel intensity criterion for the underlying thresholding. The surface roughness of the reconstruction was set to 2 μm which provided the most stable solutions in the range from 0.1 μ1 to 15 μm. Internal surfaces with surface area *A* < 1000 μm^2^ were excluded to only extract the cell surface.

### 2D imaging

Imaging of cell shape changes and hydrodynamic flow was mainly done in 2D using custom-made imaging chambers in a Zeiss Cellobserver transmission light microscope at one frame per second temporal resolution at 21 °C. A Plan-Apochromat 20x/0.8 objective was used together with an exposure time of 1 s. The cells are chosen such that the AV-axis of the oocyte visually lies in the imaging plane. Oocytes for which it was found in image analysis that the AV-axis does not lie in the imaging plane were not taken into account for further evaluation. 2D fluorescent images and most of the cells used in Fig. S3 Fig were acquired with a Leica SP5 confocal microscope with a 1.1 NA HC PL APO ×40 water immersion objective. It was equipped with a fast Z-focusing device (SuperZ Galvo stage) (Leica Microsystems).

### Cell surface tracking

Initially, each frame is Gaussian filtered and edge filtered by applying a two-dimensional Sobel filter. Next, a preliminary contour is initialised that is iteratively adjusted for the edges in the image. The active contour algorithm of the Python package scikit-image [47] is used for this step. The contour is optimised by weighting contour length, smoothness and the edges in the image.

### Surface quantification

The surface quantification is sketched in Fig 2B. The surface points are expressed in planar polar coordinates with the centre of mass as the origin. Thus different cells and time points can be compared irrespective of the position in the frame. This results in a radius function *r*(*θ*, *t*). By spatially Fourier transforming this function and cutting off high spatial frequencies, the radius function is smoothed. The AV-axis is detected by finding the points that develop from a local minimum to a local maximum back to a local minimum during the SCW as it is shown in Fig 2C and 2D. The radius function is shifted so that the animal pole lies at *θ* = 0. Only the symmetric parts are kept in the Fourier transform and therefore the symmetrised radius function is expressed as $\tilde{r}\left(\theta ,t\right)=\sum_{k=0}^{6}a_{k}\left(t\right)\cos\left(k\theta \right)$.

### Quantification of surface velocities

The radial surface velocities are determined by calculating the numerical derivative of two subsequent time steps. In order to reduce noise, the coefficients *a*_*k*_(*t*) are smoothed by cutting off high temporal frequencies. The cut off frequency is set for each order of *k* individually. The radial surface velocity is then calculated as: $v\left(\theta ,t\right)=\frac{{\tilde{r}}^{\prime }\left(\theta ,t+\Delta t\right)-{\tilde{r}}^{\prime }\left(\theta ,t\right)}{\Delta t}=\frac{\sum_{k=0}^{6}({\tilde{a}}_{k}\left(t+\Delta t\right)-{\tilde{a}}_{k}\left(t\right))\cos\left(k\theta \right)}{\Delta t}$, where ${\tilde{a}}_{k}\left(t\right)$ is the temporally smoothed Fourier coefficient for mode *k* and time point *t*.

### Flow measurement

Cytoplasmic flows are experimentally measured by particle image velocimetry (PIV) [33, 34]. In PIV each image is segmented and each segment is correlated with the subsequent image. The image segment is assumed to have flown where correlation is highest. We use the algorithm from the Python package openpiv [34]. Especially in regions with little contrast (the nucleus, outside of the oocyte) the algorithm encounters problems. We apply several sorting steps to the vertices found by the algorithm in order to identify ill-detected vertices. Different filters are applied for this task in the following order: At first a local-median filter is applied. A vector is discarded, if its strength and direction deviates too much from the surrounding flows. Next, a global value filter is applied, discarding flows that are unphysically strong. For each flow a signal to noise ratio of detection is stored that can be used to exclude flows. As we are only interested in the flows inside the oocyte, other flows can be discarded. In the end, we apply a global standard deviation filter, that means that if a flow deviates too much from the global stream pattern, it is sorted out.

### Curvature calculation from 2D data

We parametrise the rotationally symmetric surface as

$$\begin{array}{c} \vec{f}\left(\varphi ,\theta \right)=R\left(\theta \right)(\begin{array}{c} \cos\theta \cos\varphi \\ \cos\theta \sin\varphi \\ \sin\theta \end{array}), \end{array}$$ (1)

where $\theta \in [-\frac{\pi }{2},\frac{\pi }{2})$ is the polar angle defined between the *xy*-plane and the point of consideration. E.g. $\theta =-\frac{\pi }{2}$ defines the point $\vec{x}=(\begin{array}{c} 0\\ 0\\ -1 \end{array})$. *φ* ∈ [0, 2*π*) is the azimuthal angle measured between *xz*-plane and the point of consideration. The coordinates $\theta =0,\varphi =\frac{\pi }{2}$ define the point $\vec{x}=(\begin{array}{c} 0\\ 1\\ 0 \end{array})$. We then get the following entries for the Weingarten matrix:

$$\begin{array}{c} a_{\varphi \varphi }=\frac{-1-\frac{\left(\partial_{\theta }R\right)\text{tan}\theta }{R}}{\sqrt{R^{2}+{\left(\partial_{\theta }R\right)}^{2}}},\;a_{\theta \theta }=\frac{-1+\frac{\left(-{\left(\partial_{\theta }R\right)}^{2}+R\left(\partial_{\theta }\partial_{\theta }R\right)\right)}{\left(R^{2}+{\left(\partial_{\theta }R\right)}^{2}\right)}}{\sqrt{R^{2}+{\left(\partial_{\theta }R\right)}^{2}}}. \end{array}$$ (2)

From the Weingarten matrix mean curvature (*H*) and Gaussian curvature (*K*) are computed as:

$$\begin{array}{c} H=\frac{1}{2}\text{tr}\left(a\right)=\frac{a_{\varphi \varphi }+a_{\theta \theta }}{2},\;K=\det\left(a\right)=a_{\varphi \varphi }a_{\theta \theta }. \end{array}$$ (3)

More details of the calculations are given in S1 Appendix.

### Volume and surface area from 2D data

Volume and surface area can then be calculated directly from the Fourier coefficients up to *k*_*max*_ = 4 as:

V=∫ΩdΩ=∫0πdθ∫0R(θ)dR2πR2sin(θ)=2π3∫0πdθ(∑k=04cos(kθ)ak)3sin(θ)=2π3(2a03-25a02(5a2+a4)+2105a0(105a12+147a22-126a1a3+153a32-114a2a4+155a42)-215015(3861a23+8437a2a32-429a12(7a2-13a4)-9581a22a4+215015(5369a32a4+7943a2a42+777a43-286a1a3(45a2+49a4))).

For the surface area *A* we get:

A=∫∂ΩdA=∫0πdθR22πsin(θ)=2π∫0πdθ(∑k=04cos(kθ)ak)2sin(θ)=4π315(315a02-42(5a2+a4)a0+105a12+147a22+=4π315(153a32+155a42-126a1a3-114a2a4).

### Calculating internal flows from surface movement

We calculate hydrodynamic flow for a Newtonian, incompressible fluid at low Reynolds number. Reynolds number of starfish internal flows is about 2 × 10^−4^, thus we can use the Stokes equation. Even though the volume is changing over time, the assumption of local incompressibility can still be made for the fluid. For each time point the internal flows are calculated anew from the current surface movement. This is valid because the Stokes equation is time-independent, thus the past history of the flow is not required. We solve the standard form of the Stokes equation [48, 49]:

$$\begin{array}{cc} \mu {\vec{\nabla }}^{2}u_{i} & =\frac{\partial p}{\partial x_{i}} \end{array}$$ (4)

$$\begin{array}{cc} \vec{\nabla }\cdot \vec{u} & =0 \end{array}$$ (5)

with the standard no-slip boundary condition at the cell surface. Thus the movement of the cell envelope serves as boundary condition for the internal flows. Lamb has given a solution for spherical coordinates [48] that Happel and Brenner have adjusted to external flows emerging from boundary flows on a sphere [50]. The solution to the Stokes equation for the flows inside a moving spherical shell is given by:

$$\begin{array}{c} \vec{u}=\sum_{n=-\infty }^{\infty }[\vec{\nabla }\phi^{n}+\vec{\nabla }\times (\vec{r}\chi^{n})+Ar^{2}\vec{\nabla }p^{n}+B\vec{r}p^{n}], \end{array}$$ (6)

with:

$$\begin{array}{c} A=\frac{n+3}{2\mu (n+1)(2n+3)} \end{array}$$ (7)

$$\begin{array}{c} B=-\frac{n}{\mu (n+1)(2n+3)} \end{array}$$ (8)

and three fields of solid harmonic functions of order *n* that have to determined from the boundary conditions: *p*^*n*^(*r*, *θ*, *φ*), *ϕ*^*n*^(*r*, *θ*, *φ*) and *χ*^*n*^(*r*, *θ*, *φ*), where $p=\sum_{-\infty }^{\infty }p^{n}$ is the pressure field. For the flows inside a sphere of radius *a* it holds:

$$\begin{array}{c} \phi^{n}=\chi^{n}=p^{n}=0.\;n<1 \end{array}$$ (9)

In order to calculate the other orders, three fields of spherical harmonics have to be calculated from the velocity field at the surface $\vec{u}\left(a,\theta ,\varphi \right)$.

$$\begin{array}{cc} u_{r}\left(a,\theta ,\varphi \right) & =\sum_{n=0}^{\infty }X^{n}\left(\theta ,\varphi \right) \end{array}$$ (10)

$$\begin{array}{cc} -r\vec{\nabla }\cdot \vec{u}\left(a,\theta ,\varphi \right) & =\sum_{n=0}^{\infty }Y^{n}\left(\theta ,\varphi \right) \end{array}$$ (11)

$$\begin{array}{cc} \vec{r}\cdot (\vec{\nabla }\times \vec{u}\left(a,\theta ,\varphi \right)) & =\sum_{n=0}^{\infty }Z^{n}\left(\theta ,\varphi \right). \end{array}$$ (12)

They are then matched up for *n* > 0 in the following way:

$$\begin{array}{c} p^{n}\left(r,\theta ,\varphi \right)=\frac{\mu (2n+3)}{na}\frac{r^{n}}{a^{n}}(Y^{n}-\left(n-1\right)X^{n}) \end{array}$$ (13)

$$\begin{array}{cc} \phi^{n}\left(r,\theta ,\varphi \right) & =\frac{a}{2n}\frac{r^{n}}{a^{n}}((n+1)X^{n}-Y^{n}) \end{array}$$ (14)

$$\begin{array}{cc} \chi^{n}\left(r,\theta ,\varphi \right) & =\frac{1}{n(n+1)}\frac{r^{n}}{a^{n}}Z^{n}. \end{array}$$ (15)

This result has been generalised by Brenner in a perturbation ansatz for the flows outside a slightly deformed moving sphere [51]. We here give it for the flows inside the surface. If the radius for 0 < *ϵ* < 1 is given as:

$$\begin{array}{c} r\left(\theta ,\varphi \right)=a(1+\epsilon f(\theta ,\varphi )), \end{array}$$ (16)

where *f*(*θ*, *φ*) is a linear combination of spherical harmonics, then the velocity field and the pressure field can also be expanded in orders of *ϵ*:

$$\begin{array}{c} \vec{u}=\sum_{k=0}^{\infty }\epsilon^{k}{\vec{u}}^{\left(k\right)},\;p=\sum_{k=0}^{\infty }\epsilon^{k}p^{\left(k\right)}. \end{array}$$

The zeroth order is set to the velocity field of the perturbed sphere $\vec{U}\left(\theta ,\varphi \right)$:

$$\begin{array}{c} {\vec{u}}^{\left(0\right)}{|}_{r=a}=\vec{U}\left(\theta ,\varphi \right) \end{array}$$

and for higher order *k* > 0 this leads to:

$$\begin{array}{c} {\vec{u}}^{\left(k\right)}{|}_{r=a}=-\sum_{j=1}^{k}\frac{{\left(af\left(\theta ,\varphi \right)\right)}^{j}}{j!}\frac{\partial^{j}{\vec{u}}^{\left(k-j\right)}}{\partial r^{j}}{|}_{r=a} \end{array}$$ (17)

For each perturbation order the velocity field and pressure field is calculated separately as for an unperturbed sphere, from the equations given previously. By assuming rotational symmetry of the system, numerous terms in this derivation simplify. More details of the calculations are given in S1 Appendix.

### Viscosity measurement

The diffusion constant of 50 kDa fluorescently labelled dextran was determined by fluorescence correlation spectroscopy (FCS) (see [52] for characterisation of Dextrans) inside the starfish oocyte *D*_*cyt*_ and in plain water *D*_*H*20_. From Stokes-Einstein equation $D=\frac{k_{B}T}{6\pi \mu R}$ we get the ratio of viscosities as $\frac{\mu_{cyt}}{\mu_{H20}}=\frac{D_{H20}}{D_{cyt}}$.

### Modelling shape of a contractile sphere

3D simulations of the oocyte shapes were performed following Bischof *et al*. [21] using the software SurfaceMaster [53]. The surface is modelled by a triangular mesh with locally varying surface tension. Surface tension was locally set by a Gaussian shaped band $\sigma \left(\theta \right)=A\exp(-\frac{{\left(\theta -\theta_{0}\right)}^{2}}{s^{2}})$, with the amplitude *A*, position *θ*_0_ and width *s*. For each time step independently a surface Hamiltonian with the contraction band at a specific position is minimised in steady state due to overdamped dynamics of the system. The Hamiltonian reads:

$$\begin{array}{c} H=\int_{A}^{}\;dA\;(\kappa_{b}H^{2}+\sigma \left(\vec{x}\right))+\int_{A_{0}}^{}\;dA_{0}\;(\frac{K_{\alpha }}{2}\alpha^{2}+\tilde{\mu }\beta )+k_{V}{\left(V-V_{0}\right)}^{2}, \end{array}$$

where $\kappa_{b},K_{\alpha },\;\tilde{\mu }\;\text{and}\;k_{V}$ are the bending rigidity, stretch modulus, strain modulus and volume modulus respectively. They are fixed for the whole simulation. *α* and *β* are the strain invariants computed from stretch and shear of the undeformed sphere *A*_0_. *V*_0_ is the reference volume. The parameters were chosen following Bischof *et al*. [21]: Initial radius of the oocyte: 90 μm, $\tilde{\mu }=0.5\;\text{nN}\;\mu {\text{m}}^{-1}$, *K*_*α*_ = 1 nN μm^−1^, *κ*_*b*_ = 0.2 pN μm and *k*_*V*_ = 0.01 nN/μm^2^.

### Modelling surface flow of a contractile sphere

In order to model the cytoplasmic flows due to a tangential movement of the cell surface, we need to calculate the effect of a localised contraction band on the tangential surface movement. The results of these calculations are shown in Fig 5D. For this task we follow the work by Mayer *et al*. [32] who modelled the cortex of *C. elegans* as a viscoelastic medium. We also neglect the elastic contribution due to the slow dynamics of the system and thus consider a purely viscous medium. It is assumed that the microscopic restructuring of the cortical network is on timescales faster than the flow dynamics. They have shown that the viscous flow *v* in a 1D system with contraction $C(\tilde{x})$ is described by the following differential equation:

$$\begin{array}{c} -\frac{\partial C}{\partial \tilde{x}}=\frac{\partial^{2}v}{\partial {\tilde{x}}^{2}}-b^{2}v, \end{array}$$

with $\tilde{x}$ a normalised length scale and $b=\frac{L}{l}$ the ratio between the length of the system and the spatial decay length of the velocity. The contraction field $C(\tilde{x})$ thus determines the local strength of isotropic contraction and models the decrease in surface area by locally increased surface tension in our case. The boundary conditions are set to *v*(0) = *v*(*L*) = 0 and *v*′(0) = *v*′(*L*) = 0, because of symmetry of the oocyte. We solved it for a Gaussian contraction band $C\left(\tilde{x}\right)=A\exp(-\frac{{\left(\tilde{x}-{\tilde{x}}_{0}\right)}^{2}}{s^{2}})$. This equation is solved by

$$\begin{array}{c} v\left(\tilde{x}\right)=w_{1}v_{1}+w_{2}v_{2}, \end{array}$$

where $v_{1}\left(\tilde{x}\right)=\exp(b\tilde{x}),v_{2}\left(\tilde{x}\right)=\exp(-b\tilde{x})$, $w_{1}\left(\tilde{x}\right)=\frac{1}{2b}\int_{}^{\tilde{x}}\;dx^{\prime }\;\frac{\partial C(x^{\prime })}{\partial x^{\prime }}\exp\left(-bx^{\prime }\right)$ and the function $w_{2}\left(\tilde{x}\right)=-\frac{1}{2b}\int_{}^{\tilde{x}}\;dx^{\prime }\;\frac{\partial C(x^{\prime })}{\partial x^{\prime }}\exp\left(bx^{\prime }\right).$ We solved this system of equations analytically using the commercial software Mathematica and the resulting tangential flows are used as surface movement in the hydrodynamic calculations.

### Fitting centre of mass movement and contraction

Due to linearity of the Stokes equation, different contributions to the internal flows can be calculated separately. To account for CM movement during imaging rigid body movement with varying velocity along the AV-axis is added to the flows emerging from radial surface movement. The shift velocity is fitted to the experimentally observed flows for each time step individually.

Tangential surface movement is modelled by adding the flows emerging from a contraction band at position ${\tilde{x}}_{0}$ and strength *A*. The width of the contraction band is set to *s* = 0.63, which was a result of the shape simulation. The ratio of length scales is set to *b* = 3 following the result from Mayer *et al*. [32]. This is justified as we have observed that the flows emerging from the contraction band vary only little for varying *b* in the range of consideration. The parameters ${\tilde{x}}_{0}$ and *A* are found by a brute force approach. The parameter set that showed minimal root mean squared deviation between model and experiment is chosen.

Particle trajectories were calculated by integrating the experimentally obtained flow field numerically.

## Supporting information

S1 Fig Surface tracking for 24 oocytes. For 24 oocytes the surface tracking (blue) and the radius function (orange) are shown for the time point after the SCW, when maximal shape changes were observed. The animal pole is marked by a star and the AV-axis by a blue line. The difference between the smoothed radius function (fft) and its symmetrised version is small in all cases. The top left cell is the representative cell used in the main text. If the transmission mode of a confocal microscope has been used, the oil droplet from the dextran microinjection is still visible.
(TIFF)

S2 Fig Curvature kymograph for 3D data. Curvature quantities such as K2 can also be calculated for the 3D data presented in Fig 1. We see that the SCW reveals itself in a similar way as for the 2D data analysed in Fig 3H, although resolution is not as good. Moreover this particular cell is already more curved before the SCW sets in, as shown by the colour bands.
(TIFF)

S3 Fig Movement of contraction band. Width, amplitude, and position of the Gaussian shaped band of locally increased surface tension for the travelling band model as obtained from a fit of the model to the experimental data.
(TIFF)

S4 Fig Additional time points for the tangential displacement model. Additional time points for the comparison of experimental data (red arrows) and the tangential displacement model (black arrows). The model is based on radial surface movement from image analysis, fitting centre of mass movement and fitting the strength and the position of a Gaussian contraction band. The pressure field is visualised in purple to green colours together with radial surface movement (blue to red rim) that is determined from image analysis.
(TIFF)

S5 Fig Volume and surface area calculations for N = 24 cells. Dynamics of volume and surface area for 13 cells calculated from the 2D data (left panels). In contrast to the direct 3D measurements from Fig 1, now volume and surface area develop in a similar manner because they are calculated from the same contour data. Moreover the absolute changes are an overestimation because the oocytes are flattened in the imaging chambers. Similar to the 3D data from Fig 1, the surface area decreases during the SCW and shows an overshoot after the SCW. The two uppermost cells in volume (black and dark blue) still had their jelly coat, whereas the others had it removed by Actinase or HCl treatment. *t* = 0 was set to the minimal volume during the SCW in order to align different cells. Volume and surface area were normalised with respect to the maximum before the wave. The normalised data was smoothed with a Gaussian for clarity (middle panels). Box plots of the full data set (N = 24) show the relative volume and surface area losses.
(TIFF)

S1 Video 3D view of SCW of fluorescently labelled oocyte. 3D view of the fluorescence signal of the SCW on an oocyte filled with fluorescently labelled dextran. The images have been generated with Fiji.
(MP4)

S2 Video 3D reconstruction of SCW. 3D reconstruction of the surface of an oocyte imaged by fluorescence microscopy. The surface reconstruction was done with Imaris.
(MP4)

S3 Video Deformation during SCW. Video of the deformation process during the SCW recorded in the central 2D slice. This video shows the full time course of the images shown in Fig 2.
(MP4)

S4 Video Experimentally measured flows inside the starfish oocyte. Video of the flows inside the starfish oocyte during the SCW as measured by particle image velocimetry. This video shows the full time course of the images shown in Fig 6.
(MP4)

S5 Video Prediction of the radial displacement model. Video of the flows inside the starfish oocyte during the SCW as predicted by the radial displacement model compared with experimentally determined flows. This video shows the full time course of the image shown in Fig 7A.
(MP4)

S6 Video Cell with stronger tangential flow component. Transmitted light microscopy video of a cell with a strong tangential flow component during the SCW.
(MP4)

S7 Video Internal flows of the cell of S6 Video. Flow measurement of the cell presented in S6 Video performed by PIV.
(MP4)

S8 Video Prediction of the tangential displacement model. Video of the flows inside the starfish oocyte during the SCW as predicted by the tangential displacement model compared with experimentally determined flows. This video shows the full time course of the image shown in Fig 7B.
(MP4)

S9 Video Oocyte treated with blebbistatin. Both the strength of the SCW and the cytoplasmic flows is weakened by treatment with the myosin II inhibitor blebbistatin. However, the polar body is still appearing at the end of the SCW.
(MP4)

S10 Video Flows inside blebbistatin treated oocyte. PIV-analysis of the flows during the SCW of the oocyte in S9 Video treated with blebbistatin. The flows are much weaker than for the wild type cell.
(MP4)

S11 Video Actin distribution during the SCW by labelling with utrophin. The internal distribution of actin in the oocyte is imaged by labelling it with utrophin. One clearly sees the accumulation of actin at the position of the polar body.
(MP4)

S12 Video Cortical actomyosin localization during the SCW. During the SCW, the dynamical accumulation of actomyosin in the cortex can be observed by fluorescent labelling of the Myosin II heavy chain with GFP (magenta) and actin by Utrophin-mCherry (cyan). Both move together with the SCW along the surface of the oocyte, and most likely also lead to tangential actomyosin flow, as predicted by our model.
(AVI)

S1 Appendix Detailed calculations. Detailed calculations for the flows inside a slightly deformed sphere emerging from surface movement and of the curvatures of a rotationally symmetric object.
(PDF)
