# Supplementary figures and images for: Cytoplasmic flows in starfish oocytes are fully determined by cortical contractions
Source: PLoS Comput Biol. 2018 Nov 15;14(11):e1006588. doi: 10.1371/journal.pcbi.1006588 (PMC6264906; doi:10.1371/journal.pcbi.1006588)

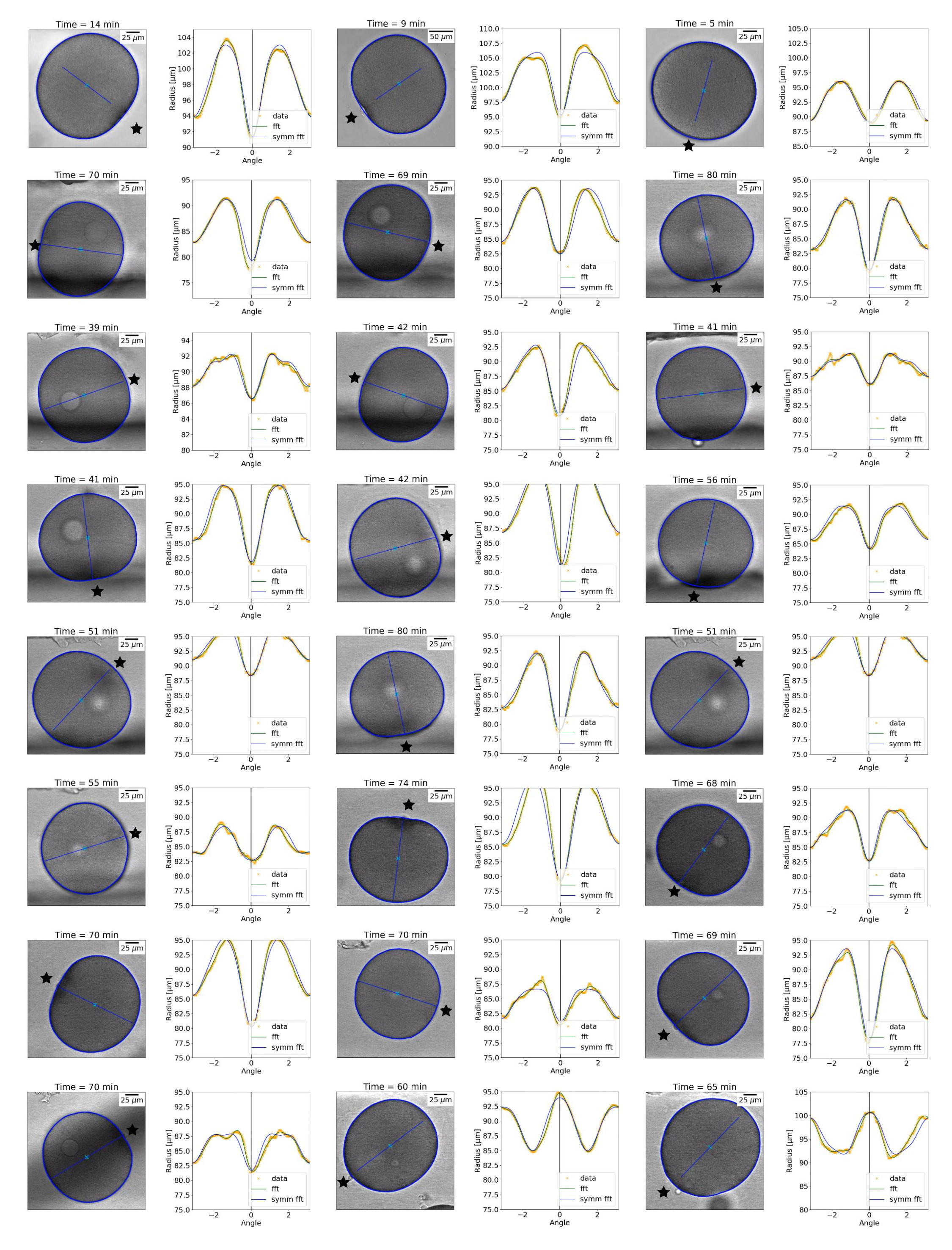

Supplement: S1 Fig — For 24 oocytes the surface tracking (blue) and the radius function (orange) are shown for the time point after the SCW, when maximal shape changes were observed. The animal pole is marked by a star and the AV-axis by a blue line. The difference between the smoothed radius function (fft) and its symmetrised version is small in all cases. The top left cell is the representative cell used in the main text. If the transmission mode of a confocal microscope has been used, the oil droplet from the dextran microinjection is still visible. (TIFF) [file pcbi.1006588.s001.tiff]

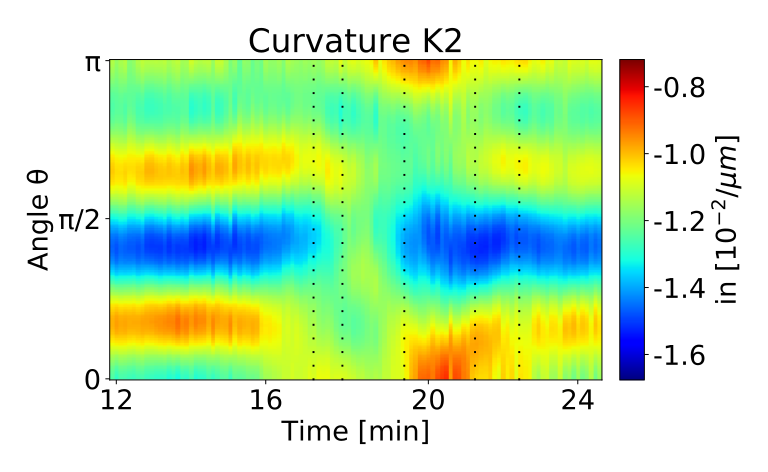

Supplement: S2 Fig — Curvature quantities such as K2 can also be calculated for the 3D data presented in Fig 1. We see that the SCW reveals itself in a similar way as for the 2D data analysed in Fig 3H, although resolution is not as good. Moreover this particular cell is already more curved before the SCW sets in, as shown by the colour bands. (TIFF) [file pcbi.1006588.s002.tiff]

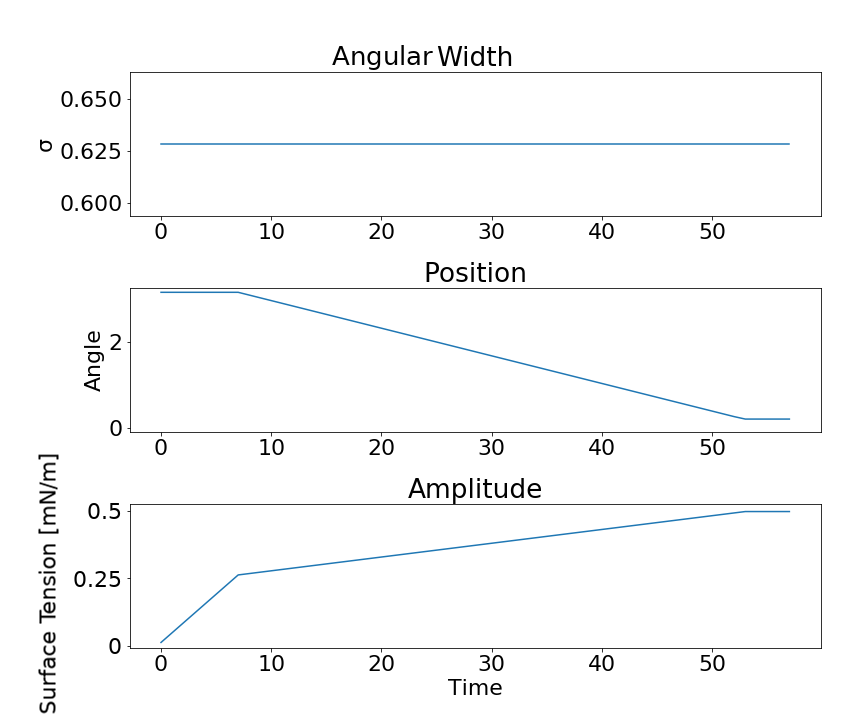

Supplement: S3 Fig — Width, amplitude, and position of the Gaussian shaped band of locally increased surface tension for the travelling band model as obtained from a fit of the model to the experimental data. (TIFF) [file pcbi.1006588.s003.tiff]

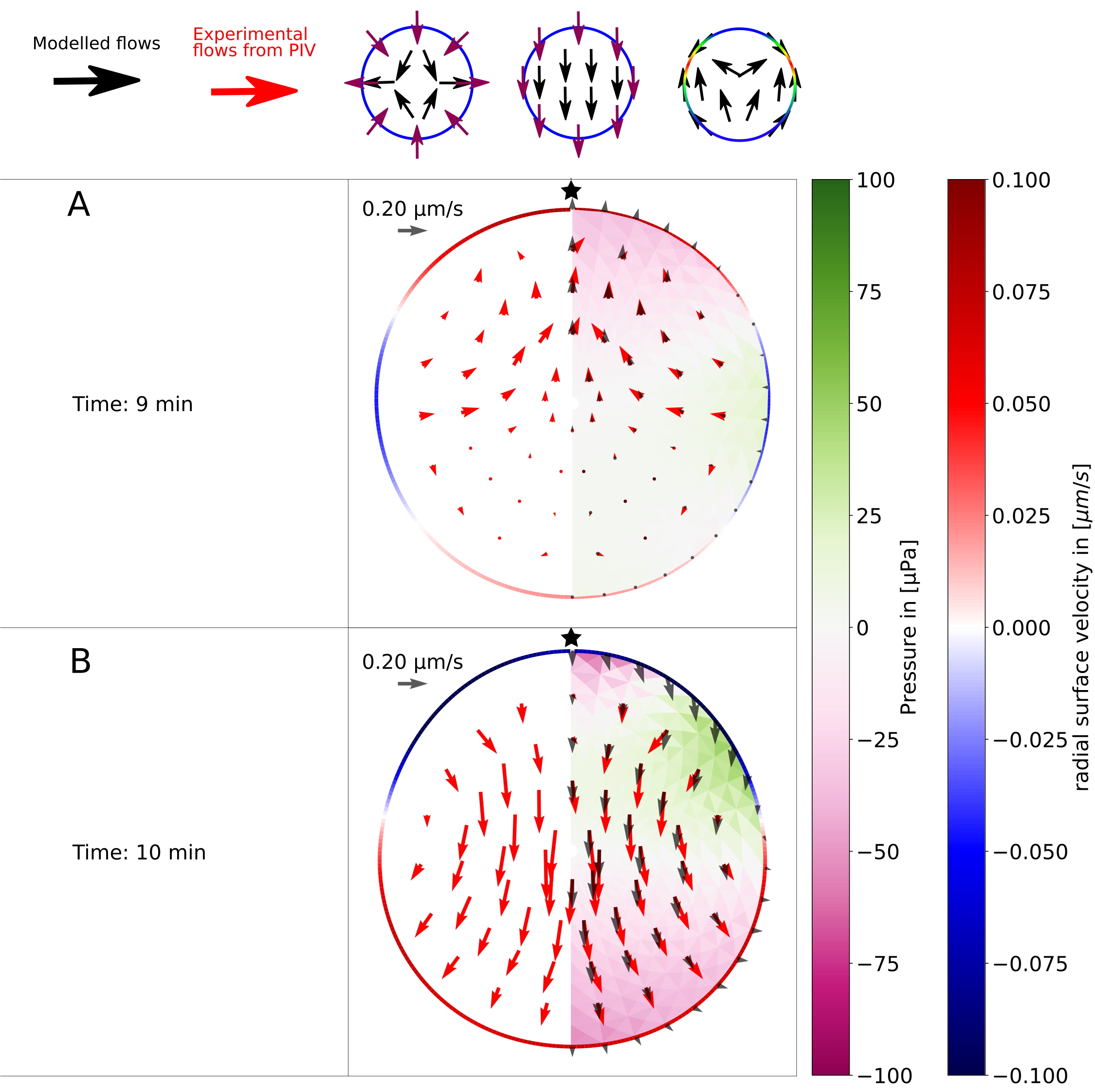

Supplement: S4 Fig — Additional time points for the comparison of experimental data (red arrows) and the tangential displacement model (black arrows). The model is based on radial surface movement from image analysis, fitting centre of mass movement and fitting the strength and the position of a Gaussian contraction band. The pressure field is visualised in purple to green colours together with radial surface movement (blue to red rim) that is determined from image analysis. (TIFF) [file pcbi.1006588.s004.tiff]

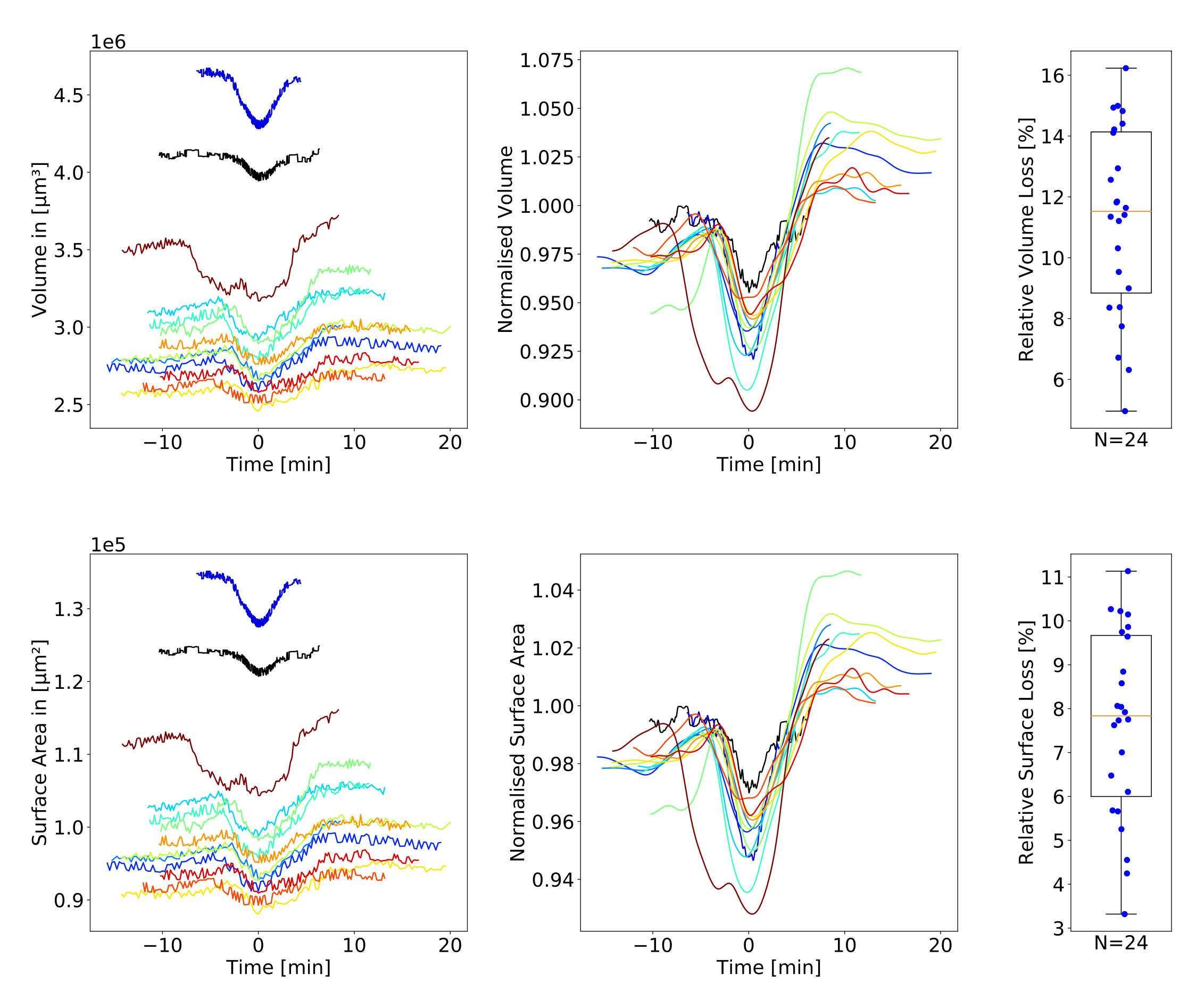

Supplement: S5 Fig — Dynamics of volume and surface area for 13 cells calculated from the 2D data (left panels). In contrast to the direct 3D measurements from Fig 1, now volume and surface area develop in a similar manner because they are calculated from the same contour data. Moreover the absolute changes are an overestimation because the oocytes are flattened in the imaging chambers. Similar to the 3D data from Fig 1, the surface area decreases during the SCW and shows an overshoot after the SCW. The two uppermost cells in volume (black and dark blue) still had their jelly coat, whereas the others had it removed by Actinase or HCl treatment. t = 0 was set to the minimal volume during the SCW in order to align different cells. Volume and surface area were normalised with respect to the maximum before the wave. The normalised data was smoothed with a Gaussian for clarity (middle panels). Box plots of the full data set (N = 24) show the relative volume and surface area losses. (TIFF) [file pcbi.1006588.s005.tiff]
