# Supplementary material for: Cytoplasmic flows in starfish oocytes are fully determined by cortical contractions
Source: PLoS Comput Biol. 2018 Nov 15;14(11):e1006588. doi: 10.1371/journal.pcbi.1006588 (PMC6264906; doi:10.1371/journal.pcbi.1006588)
Supplement: S1 Appendix — Detailed calculations for the flows inside a slightly deformed sphere emerging from surface movement and of the curvatures of a rotationally symmetric object. (PDF) [file pcbi.1006588.s018.pdf]

Cytoplasmic flows in starfish oocytes are fully determined by cortical contractions - Supplemental Text

Nils Klughammer[1,¤a], Johanna Bischof[2,¤b], Nikolas D. Schnellbächer[1], Andrea Callegari[2], Péter Lénárt[2,¤c], Ulrich S. Schwarz[1*]

1 Institute for Theoretical Physics and BioQuant, Heidelberg University, Philosophenweg 19, 69120 Heidelberg, Germany
2 Cell Biology and Biophysics Unit, European Molecular Biology Laboratory (EMBL), Meyerhofstrasse 1, 69117 Heidelberg, Germany

¤a Current address: Department of Bionanoscience, Kavli Institute of Nanoscience, Delft University of Technology, Van der Maasweg 9, 2628 CJ Delft, The Netherlands
¤b Current address: Center for Regenerative and Developmental Biology, Tufts University, 200 Boston Ave Suite 4600, Medford, MA 02155, USA
¤c Current address: Max-Planck Institute for Biophysical Chemistry, Am Fassberg 11, 37077 Göttingen, Germany

* Corresponding author: schwarz@thphys.uni-heidelberg.de

Solution of the Stokes equation inside a Slightly Deformed Sphere

Solid Harmonic Functions

As we are aiming to solve Laplace's equation together with another condition, we should have a short look on functions solving Laplace's equation: Solid Harmonic Functions also known as Solid Spherical Harmonics. Apart from forming a complete set, solid harmonic functions have additional useful properties. Let $f^n(r,\theta,\varphi)$ be a solid harmonic function of order n, then it holds:

$$\vec{\nabla}^2 f^n(r,\theta,\varphi) = \left(\frac{1}{r}\frac{\partial^2}{\partial r^2}r - \frac{\hat{l}^2}{r^2}\right) f^n(r,\theta,\varphi) = 0, \qquad r \neq \mathbf{0}.$$

They are formed using spherical harmonics Y_l^m, being the eigenfunctions of the angular momentum operator $\hat{l}^2$. Thus solid harmonic functions can be separated in two forms: Regular R_l^m and irregular ones I_l^m of order l defined by:

$$R_l^m(\mathbf{r}) \equiv \sqrt{\frac{4\pi}{2l+1}}\, r^l Y_l^m(\theta,\varphi)$$

and

$$I_l^m(\mathbf{r}) \equiv \sqrt{\frac{4\pi}{2l+1}}\, \frac{Y_l^m(\theta,\varphi)}{r^{l+1}},$$

where Recah's normalisation was used. The choice of a special normalisation is not important for us though.

In most derivations only the algebraic order of the radial dependence is important for us, therefore we introduce a shorthand notation. This is useful, because all equations we are dealing with, are linear. n is called the order of the solid harmonic function and is the exponent of the radial part:

$$f^n(r,\theta,\varphi) = r^n S^n(\theta,\varphi) = \sum_{m=-n}^{n} a_m R_n^m(r,\theta,\varphi) \qquad n \geq 0$$

$$f^n(r,\theta,\varphi) = r^n S^n(\theta,\varphi) = \sum_{m=-(n-1)}^{(n-1)} b_m I_{-n-1}^m(r,\theta,\varphi) \qquad n \leq -1.$$

Here scalar coefficients a_m and b_m have been introduced, that are not set to a specific value. Additionally we have introduced the short hand notation for a general linear

combination of spherical harmonics S^n differently defined for $n \geq 0$ and $n \leq -1$. This enables us to perform many calculations in a short hand notation without separating between regular and irregular solid harmonic functions.

Using this notation an expression like:

$$\sum_{n=-\infty}^{\infty} f^n$$

means that it is summed over all polynomial orders in r^n with all corresponding spherical harmonic functions, each spherical harmonic having its own coefficient. Thus a sum like the above one resembles a complete solution to Laplace's equation. In this notation prefactors can be absorbed in the sum, so one has to be careful in these cases.

Having defined spherical harmonic functions, we next state some handy equalities that will be used in the remainder of the chapter.

For the derivative in radial direction we find:

$$\left(\vec{r} \cdot \vec{\nabla}\right) f^n = \left(r \frac{\partial}{\partial r}\right) f^n = \left(r \frac{\partial}{\partial r}\right) r^n S_n(\theta, \varphi) = n r^n S_n(\theta, \varphi) = n f^n. \tag{1}$$

Additionally it holds that:

$$\vec{\nabla}^2 \left(\vec{r} f^n\right) = 2 \vec{\nabla} f^n. \tag{2}$$

as:

$$\begin{aligned}
\left(\vec{\nabla}^2 \left(\vec{r} f^n\right)\right)_j &= \sum_{i=1}^{3} \left(\frac{\partial^2}{\partial x_i^2} \left(x_j f^n\right)\right) \\
&= \sum_{i=1}^{3} \frac{\partial}{\partial x_i} \left(\delta_{ij} f^n + x_j \frac{\partial f^n}{\partial x_i}\right) \\
&= \frac{\partial f^n}{\partial x_j} + \sum_{i=1}^{3} \delta_{ij} \frac{\partial f^n}{\partial x_i} + x_j \underbrace{\sum_{i=1}^{3} \frac{\partial^2 f^n}{\partial x_i^2}}_{0} \\
&= 2 \frac{\partial f^n}{\partial x_j} = 2 \left(\vec{\nabla} f^n\right)_j,
\end{aligned}$$

where in the second to last line we used the property of solid harmonic functions satisfying Laplace equation.

Terms like $\vec{\nabla}^2 \left(r^m f^n\right)$ can be simplified due to the properties of solid harmonics:

$$\vec{\nabla}^2 \left(r^m f^n\right) = m \left(m + 2n + 1\right) r^{m-2} f^n. \tag{3}$$

This can be shown by using that any solid harmonic of order $f^n(r,\theta,\varphi)$ can be split up in a radial part and a linear combination of spherical harmonics of degree n which we will denote as S^n

$$\begin{aligned}\vec{\nabla}^2\left(r^m f^n\right) &= \vec{\nabla}^2\left(r^{m+n}S^n\right)\\ &= \frac{1}{r}\frac{\partial^2}{\partial r^2}r\left(r^{m+n}S^n\right) - \frac{\hat{l}^2}{r^2}\left(r^{m+n}S^n\right)\\ &= \frac{1}{r}(m+n)(m+n+1)r^{(m+n)-1}S^n - n(n+1)r^{m+n-2}S^n\\ &= \left[m(m+n+1)+nm+n(n+1)-n(n+1)\right]r^{m+n-2}S^n\\ &= m(m+2n+1)r^{m-2}f^n,\end{aligned}$$

where in the second line we have split up the Laplace operator in three dimensions in its angular and radial part and then have used that S^n is an eigenfunction to the angular momentum operator $\hat{l}^2$.

As the radial dependence of a solid harmonic function is completely determined by its order, we can rewrite a solid harmonic function evaluated at a certain radius. Here we pull out the dependence on the evaluation radius to obtain the full radial dependence in the solid harmonic:

$$f^n(r,\theta,\varphi)\big|_{r=a} = \left[r^n S^n(\theta,\varphi)\right]_{r=a} = a^n\frac{r^n}{r^n}S^n(\theta,\varphi) = \left(\frac{a}{r}\right)^n f^n(r,\theta,\ \varphi). \tag{4}$$

Homogeneous Solution

After having set the basis with solid harmonic functions we now start to solve Laplace's equation together with continuity equation using what we have learned about solid harmonic functions. At first the flow field is rewritten. Next, for each term it is shown independently that it can be described using spherical harmonic functions by applying continuity equation.

As a reminder, we are looking for a flow field $\vec{u}(\vec{x})$ satisfying the following two equations:

$$\vec{\nabla}^2\vec{u} = 0, \qquad \vec{\nabla}\cdot\vec{u} = 0. \tag{5}$$

First of all, we rewrite the flow field. The form seems more complicated at first sight but has some advantages:

In components we start with:

$$
\begin{aligned}
-\left(\vec{r}\times\left(\vec{\nabla}\times\vec{u}\right)\right)_i =& -\varepsilon_{ijk}x_j\varepsilon_{klm}\partial_l u_m \\
=&\varepsilon_{ijk}\varepsilon_{klm}\left(\delta_{jl}u_m - \partial_l\left(x_j u_m\right)\right) \\
=&\varepsilon_{ijk}\varepsilon_{kjm}u_m + \left(\delta_{im}\delta_{jl} - \delta_{il}\delta_m j\right)\partial_l\left(x_j u_m\right) \\
=& -2\delta_{im}u_m + \partial_j\left(x_j u_i\right) - \partial_i\left(x_j u_j\right) \\
=& -2u_i + 3u_i + x_j\frac{\partial u_i}{\partial x_j} - \partial_i\left(x_j u_j\right) \\
=&u_i + x_j\frac{\partial u_i}{\partial x_j} - \partial_i\left(x_j u_j\right) \\
=&\left(\vec{u} + (\vec{r}\cdot\vec{\nabla})\vec{u} - \vec{\nabla}\left(\vec{r}\cdot\vec{u}\right)\right)_i .
\end{aligned}
$$

We end up with three terms that will be discussed in detail afterwards:

$$
\vec{u} = -\underbrace{\left(\vec{r}\cdot\vec{\nabla}\right)\vec{u}}_{1} + \underbrace{\vec{\nabla}\left(\vec{r}\cdot\vec{u}\right)}_{2} - \underbrace{\vec{r}\times\left(\vec{\nabla}\times\vec{u}\right)}_{3} . \tag{6}
$$

With this formulation in the end only two instead of three functions need to be determined. As $\vec{u}$ obeys Laplace's equation in each component, it can be expanded to solid harmonic functions as described before. Therefore we are able to compare orders of the radial part: If $u_i = u_i^n$ is of order n, all three terms on the right hand side have to match this order of n.

The first term can be easily absorbed in the left hand side by applying the rule for harmonic functions $\left(\vec{r}\cdot\vec{\nabla}\right)u_i^n = nu_i^n$ as shown in equation (1). Therefore it can be put to the left hand side giving a prefactor of $(n+1)$ for the nth order of u_i.

For the second term it holds that $\vec{\nabla}^2\left(\vec{r}\cdot\vec{u}\right) = 0$, therefore this expression can be expanded in solid harmonics too. This comes from:

$$
\vec{\nabla}^2\left(\vec{r}\cdot\vec{u}\right) = \underbrace{\vec{\nabla}^2\vec{r}}_{0} + 2\left(\vec{\nabla}\cdot\vec{r}\right)\underbrace{\left(\vec{\nabla}\cdot\vec{u}\right)}_{0} + \underbrace{\vec{\nabla}^2\vec{u}}_{0} = 0,
$$

where the conditions on $\vec{u}$ from the homogeneous Stokes equation are applied and it is used that the Laplacian of the position vector vanishes.

We choose a solid harmonic function $\phi^n(r,\theta,\varphi) = \vec{r}\cdot\vec{u}^{n-1}$ to rewrite this term. For matching up orders of r^n in the equation, we have to choose the order of ϕ to be $(n+1)$, as the derivative of a solid harmonic of order n is a solid harmonic of order $n-1$.

For the discussion of the third term in equation (6) we start by rewriting the Lapla-

cian of any differentiable vector field $\vec{w}$ in the following way:

$$\vec{\nabla}^2\vec{w} = -\vec{\nabla} \times \left(\vec{\nabla} \times \vec{w}\right) + \vec{\nabla}(\vec{\nabla} \cdot \vec{w}). \tag{7}$$

By applying the two conditions from the Stokes equation we thus obtain for the velocity field $\vec{u}$:

$$0 = \vec{\nabla}^2\vec{u} = -\vec{\nabla} \times \left(\vec{\nabla} \times \vec{u}\right) + \vec{\nabla}\,\underbrace{(\vec{\nabla} \cdot \vec{u})}_{0}. \tag{8}$$

Therefore we get that $\vec{\nabla} \times \left(\vec{\nabla} \times \vec{u}\right) = 0$. The rightmost term of equation (8) term is zero because of continuity equation (5). As the curl of $\vec{\nabla} \times \vec{u} = 0$, we can find a scalar potential function $\chi(\vec{x})$ with the property:

$$\vec{\nabla}\chi = \vec{\nabla} \times \vec{u}.$$

This is valid if the domain in consideration is simply connected, roughly meaning it has no holes, which for our applications is the case. χ obeys Laplace's equation: $\vec{\nabla}^2\chi = 0$, which we will show now:

$$\vec{\nabla}^2\chi = \sum_{i=1}^{3} \frac{\partial^2\chi}{\partial x_i^2} = \sum_{i=1}^{3} \frac{\partial}{\partial x_i}\left(\frac{\partial \chi}{\partial x_i}\right) = \frac{\partial}{\partial x_i}\varepsilon_{ijk}\frac{\partial}{\partial x_j}u_k = \underbrace{\varepsilon_{ijk}}_{\text{antisymmetric}}\underbrace{\frac{\partial}{\partial x_i}\frac{\partial}{\partial x_j}u_k}_{\text{symmetric}} = 0,$$

where we used the definition that the gradient of χ equals the rotation of the flow field $\vec{u}$. Additionally we used that ε is antisymmetric in i, j and the derivatives are symmetric in exchange of the order of differentiation, as we assume $\vec{u}$ to be continuously differentiable. Therefore the whole expression must equal to zero.

Therefore $\chi(r, \theta, \varphi)$ is a harmonic function and can be expanded in orders of Solid harmonics as $\chi = \sum_{n=-\infty}^{\infty} \chi^n$.

Replacing the three terms in equation (6) with the corresponding orders of $\phi(r, \theta, \varphi)$ and $\chi(r, \theta, \varphi)$ we end up with:

$$(n+1)\vec{u}^n = \vec{\nabla}\phi^{n+1} - \vec{r} \times \left(\vec{\nabla}\chi^n\right).$$

We can now absorb the prefactor of $(n+1)$ to the linear combination of the solid harmonics on the left and sum all orders up so that a general solution for a flow field

obeying the homogeneous Stokes equation can be written as:

$$\vec{u} = \sum_{n=-\infty}^{\infty} \left[\vec{\nabla}\phi^n - \vec{r} \times \left(\vec{\nabla}\chi^n \right) \right], \tag{9}$$

where $\phi^n(r,\theta,\varphi)$ and $\chi^n(r,\theta,\varphi)$ just need to be solid harmonic functions of order n. This vector field $\vec{u}$ then satisfies both conditions in the homogeneous Stokes equation as we will show in the following lines.

$\vec{u}$ satisfies Laplace's equation simply because it is a linear combination of solid harmonics. $\vec{\nabla} \cdot \vec{u} = 0$ because

$$\vec{\nabla} \cdot \vec{\nabla}\phi^n = \vec{\nabla}^2 \phi^n = 0$$

and

$$\vec{\nabla} \cdot \vec{r} \times \left(\vec{\nabla}\chi^n \right) = \varepsilon_{ijk}\partial_i \left(x_j \partial_k \chi^n \right) = \varepsilon_{ijk}\delta_{ij}\partial_k\chi^n + \varepsilon_{ijk}x_j\partial_i\partial_k\chi^n = 0$$

because of the contraction of the antisymmetric tensor ε_{ijk} with the symmetric ones δ_{ij} and $\partial_i\partial_k\chi^n$ which both result to zero. Using the same argumentation $\vec{r} \times \left(\vec{\nabla}\chi^n \right)$ can be rewritten to $-\vec{\nabla} \times (\vec{r}\chi^n)$:

$$\varepsilon_{ijk}x_i(\partial_j\chi^n) = \varepsilon_{ijk}[\partial_j(x_i\chi^n) - \underbrace{(\partial_j x_i)}_{\delta_{ij}}\chi^n] = \varepsilon_{ijk}\partial_j(x_i\chi^n)$$

Inhomogeneous Solution

In order to get the full solution of the inhomogeneous Stokes equations, we need to find one particular solution of this system of equations and add it to the homogeneous solution (9) found in the previous section. Our starting point hence are the inhomogeneous Stokes equations:

$$\mu\vec{\nabla}^2 u_i = \frac{\partial p}{\partial x_i}$$
$$\vec{\nabla} \cdot \vec{u} = 0.$$

At first we observe that the pressure field p satisfies Laplace's equation, too:

$$\vec{\nabla}^2 p = \left(\vec{\nabla} \cdot \vec{\nabla} \right) p = \mu\vec{\nabla} \cdot \left(\vec{\nabla}^2 \vec{u} \right) = \mu\vec{\nabla}^2 \underbrace{\vec{\nabla} \cdot \vec{u}}_{0} = 0,$$

where we only used the interchangeability of partial derivatives for the continuously

differentiable function $\vec{u}$. Therefore also the pressure field $p(r, \theta, \varphi)$ can be expanded in solid harmonics as

$$p = \sum_{n=-\infty}^{\infty} p^n.$$

This is important, as it means that we can also find the inhomogeneous solution order by order. Lamb [1, § 336] and Happel and Brenner [2, § 3.2] have given two slightly different solutions. We will stick to the one given by Happel and Brenner for the $(n+1)$th order of $\vec{u}$ from the ansatz

$$\vec{u}^{n+1} = Ar^2 \vec{\nabla} p^n + B\vec{r}p^n. \tag{10}$$

The constants A and B have to be determined from plugging this ansatz into the inhomogeneous Stokes equations. The solution is given by:

$$A = \frac{n+3}{2\mu(n+1)(2n+3)} \tag{11}$$

$$B = -\frac{n}{\mu(n+1)(2n+3)} \tag{12}$$

In the next section we show the particular steps undertaken for the determination of A and B. At first we plug it into the Poisson equation $\mu\vec{\nabla}^2\vec{u} = \vec{\nabla}p$:

$$\mu\vec{\nabla}^2\vec{u}^{n+1} = \mu A\vec{\nabla}^2 \left(r^2 \underbrace{\vec{\nabla}p^n}_{\mathcal{O}(p^{n-1})} \right) + \mu B\vec{\nabla}^2 \left(\vec{r}p^n\right).$$

Using the properties of solid harmonic functions given in the equations (3) and (2), this can be rewritten to:

$$\mu\vec{\nabla}^2\vec{u}^{n+1} = \mu A \; 2\left(2 + 2(n-1) + 1\right) \vec{\nabla}p^n + \mu B 2\vec{\nabla}p^n \overset{!}{=} \vec{\nabla}p^n$$

In the last step we have put in the condition that this ansatz solves our inhomogeneous equations. By rearranging we get the condition:

$$(2n+1)A + B = \frac{1}{2\mu}.$$

Additionally the ansatz has to fulfil the incompressibility condition:

$$
\begin{aligned}
0 = \vec{\nabla} \cdot \vec{u}^{n+1} &= A(\vec{\nabla} r^2) \cdot \vec{\nabla} p^n + Ar^2 \underbrace{\vec{\nabla}^2 p^n}_{=0} + B \underbrace{\left(\vec{\nabla} \cdot \vec{r}\right)}_{=3} p^n + B \left(\vec{r} \cdot \vec{\nabla}\right) p^n \\
&= 2A \left(\vec{r} \cdot \vec{\nabla}\right) p^n + 3Bp^n + B \left(\vec{r} \cdot \vec{\nabla}\right) p^n \\
&= n \left(2A + B\right) p^n + 3Bp^n = (2nA + (3+n)B)p^n \stackrel{!}{=} 0
\end{aligned}
$$

Here property (1) was used. In total these two conditions

$$
(2n+1)A + B = \frac{1}{2\mu} \qquad\qquad 2nA + (n+3)B = 0
$$

are solved by:

$$
A = \frac{n+3}{2\mu(n+1)(2n+3)} \qquad\qquad B = -\frac{n}{\mu(n+1)(2n+3)}.
$$

For the full solution of the Stokes equation in spherical coordinates, we therefore obtain the sum of the homogeneous solution (9) with the inhomogeneous solution:

$$
\vec{u} = \sum_{n=-\infty}^{\infty} \left[\vec{\nabla}\phi^n + \vec{\nabla} \times (\vec{r}\chi^n) + Ar^2\vec{\nabla}p^n + B\vec{r}p^n\right]. \tag{13}
$$

Flows inside a Moving Spherical Boundary

This general solution needs to be adjusted to specific boundary conditions. Happel and Brenner [2] show how to do this for several cases. We will only focus on one case: The flow inside a sphere of radius a with the velocity field given on the shell. In a next step following Brenner in [3] we will generalise these results to slightly deformed spheres. This path becomes clear, as we want to describe the flows inside the starfish oocyte during the surface contraction wave. Using the approach by Happel and Brenner, finding the fields ϕ, χ and p in the whole domain can be reduced to determining three fields of spherical harmonic functions X, Y and Z defined only on the surface of the domain. In this derivation no-slip boundary condition is assumed. In principle this means, that flows close to a moving boundary have to follow the boundaries perfectly. This simplification is most important for us, because the deformation of the surface is one of the quantities we can obtain from image analysis. Additionally the surface movement thus sets the boundary conditions needed to give a specific solution to the Stokes equation.

In order to decouple the different fields, at first we have a look at the radial compo-

nent of $\vec{u}$. By applying the curl in spherical coordinates we see that the term $\vec{\nabla}\times(\vec{r}\chi^n)$ drops out as its radial component vanishes. The other terms simply become:

$$u_r = \sum_{n=-\infty}^{\infty} \left[\partial_r \phi^n + Ar^2 \partial_r p^n + Brp^n\right]$$

Using the property (1) of harmonic functions and plugging in A and B we can further simplify this equation to:

$$u_r = \sum_{n=-\infty}^{\infty} \left[\partial_r \phi^n + Arnp^n + Brp^n\right] = \sum_{n=-\infty}^{\infty} \left[\frac{n}{r}\phi^n + \frac{n}{2\mu(2n+3)} rp^n\right]$$

We can rewrite this further by differentiation and multiplication with r to:

$$r\frac{\partial}{\partial r} u_r = \left(\vec{r}\cdot\vec{\nabla}\right) u_r = \sum_{n=-\infty}^{\infty} \left[\frac{n(n-1)}{r}\phi^n + \frac{n(n+1)}{2\mu(2n+3)} rp^n\right],$$

where we have applied (1) again in order to simplify the result.

Next we separate the field χ from the full solution (13). Therefore we take the curl of the flow field and multiply it with $\vec{r}$ in order to extract the radial contribution to the the curl.

$$\vec{r}\cdot\left(\vec{\nabla}\times\vec{u}\right) = \sum_{n=-\infty}^{\infty} n(n+1)\chi^n$$

In order to prove the equation above, we start by expanding the expression in $\vec{u}$ and then look at each term separately.

$$\begin{aligned}\vec{r}\cdot\left(\vec{\nabla}\times\vec{u}\right) = \sum_{n=-\infty}^{\infty} & \underbrace{\vec{r}\cdot\left(\vec{\nabla}\times\left(\vec{\nabla}\phi^n\right)\right)}_{1} + \underbrace{\vec{r}\cdot\left(\vec{\nabla}\times\left(\vec{\nabla}\times\left(\vec{r}\chi^n\right)\right)\right)}_{2} \\ & + \underbrace{\vec{r}\cdot\left(\vec{\nabla}\times\left(Ar^2\vec{\nabla}p^n\right)\right)}_{3} + \underbrace{\vec{r}\cdot\left(\vec{\nabla}\times\left(B\vec{r}p^n\right)\right)}_{4}\end{aligned}$$

Term 1 is zero because ϕ^n is twice differentiable and thus the order of differentiation can be exchanged.

Term 2:

$$
\begin{aligned}
\vec{r}\cdot\left(\vec{\nabla}\times\left(\vec{\nabla}\times(\vec{r}\chi^n)\right)\right) =& \vec{r}\cdot\left(\vec{\nabla}\left(\vec{\nabla}\cdot(\vec{r}\chi^n)\right)-\vec{\nabla}^2(\vec{r}\chi^n)\right)\\
\overset{(2)}{=}& \vec{r}\cdot\left(\vec{\nabla}\left(3\chi^n+\left(\vec{r}\cdot\vec{\nabla}\right)\chi^n\right)-2\vec{\nabla}\chi^n\right)\\
\overset{(1)}{=}& \vec{r}\cdot\left(\vec{\nabla}\chi^n+n\chi^n\right)\\
=& r\vec{e}_r\cdot\vec{\nabla}(n+1)\chi^n\\
=& (n+1)r\frac{\partial}{\partial r}\chi^n\\
=& n(n+1)\chi^n
\end{aligned}
$$

Term 3:

$$
\begin{aligned}
\vec{r}\cdot\left(\vec{\nabla}\times\left(Ar^2\vec{\nabla}p^n\right)\right) =& A\vec{r}\cdot\left(r^2\underbrace{\vec{\nabla}\times\left(\vec{\nabla}p^n\right)}_{=0}+(\vec{\nabla}r^2)\cdot\vec{\nabla}p^n\right)\\
=& A\vec{r}\cdot\underbrace{\left(2\vec{r}\times\vec{\nabla}p^n\right)}_{\perp\vec{r}}=0
\end{aligned}
$$

Term 4:

$$
\vec{r}\cdot\left(\vec{\nabla}\times(B\vec{r}p^n)\right)=B\vec{r}\cdot\left(\underbrace{\left(\vec{\nabla}p^n\right)\times\vec{r}}_{\perp\vec{r}}+p^n\underbrace{\vec{\nabla}\times r}_{0}\right)=0.
$$

In order to express all quantities with respect to the velocity field given on the sphere, we switch from $\vec{u}(r,\theta,\varphi)$ to $\vec{u}(a,\theta,\varphi)$. Here the a is the radius of the sphere. In the next section we will show that:

$$
u_r\Big|_{r=a} = u_r(a,\theta,\varphi) \tag{14}
$$

$$
r\frac{\partial u_r}{\partial r}\Bigg|_{r=a} = -r\vec{\nabla}\cdot\vec{u}(a,\theta,\varphi) \tag{15}
$$

$$
\vec{r}\cdot\left(\vec{\nabla}\times\vec{u}\right)\Big|_{r=a} = \vec{r}\cdot\left(\vec{\nabla}\times\vec{u}(a,\theta,\varphi)\right). \tag{16}
$$

As the divergence of $\vec{u}$ vanishes due to the continuity equation, we can add a zero in order to rewrite this expression according to our purpose:

$$
\left(\vec{r}\cdot\vec{\nabla}\right)u_r=\left(\vec{r}\cdot\vec{\nabla}\right)u_r-r\vec{\nabla}\cdot\vec{u}
$$

The reason for this trick becomes clear, when we look at this quantity at the radius

a of our sphere of interest, because in the end we only want to plug in the velocity profile at this certain radius:

$$
\begin{aligned}
& a\frac{\partial u_r}{\partial r}\bigg|_{r=a} \\
&= \left[\left(\vec{r}\cdot\vec{\nabla}\right)u_r - r\vec{\nabla}\cdot\vec{u}\right]_{r=a} \\
&= \left[r\frac{\partial u_r}{\partial r} - r\left(\frac{1}{r^2}\frac{\partial\left(r^2 u_r\right)}{\partial r} + \frac{1}{r\sin\theta}\frac{\partial}{\partial\theta}\sin\theta u_\theta + \frac{1}{r\sin\theta}\frac{\partial u_\varphi}{\partial\varphi}\right)\right]_{r=a} \\
&= \left[r\frac{\partial u_r}{\partial r} - \frac{1}{r}\left(2ru_r + r^2\frac{\partial u_r}{\partial r}\right) - r\left(\frac{1}{r\sin\theta}\frac{\partial}{\partial\theta}\sin\theta u_\theta + \frac{1}{r\sin\theta}\frac{\partial u_\varphi}{\partial\varphi}\right)\right]_{r=a} \\
&= \left[-2u_r - r\left(\frac{1}{r\sin\theta}\frac{\partial}{\partial\theta}\sin\theta u_\theta + \frac{1}{r\sin\theta}\frac{\partial u_\varphi}{\partial\varphi}\right)\right]_{r=a} \\
&= -\,2u_r(a,\theta,\varphi) - \left[r\left(\frac{1}{r\sin\theta}\frac{\partial}{\partial\theta}\sin\theta u_\theta + \frac{1}{r\sin\theta}\frac{\partial u_\varphi}{\partial\varphi}\right)\right]_{r=a} \\
&= -\left[r\left(\frac{1}{r^2}\frac{\partial\left(r^2 u_r(a,\theta,\varphi)\right)}{\partial r} + \frac{1}{r\sin\theta}\frac{\partial}{\partial\theta}\sin\theta u_\theta + \frac{1}{r\sin\theta}\frac{\partial u_\varphi}{\partial\varphi}\right)\right]_{r=a} \\
&= -\,a\vec{\nabla}\cdot\vec{u}(a,\theta,\varphi)
\end{aligned}
$$

Now both sides can be divided by a and multiplied with r so that we obtain the wanted result.

On the other hand

$$\vec{r}\cdot\left(\vec{\nabla}\times\vec{u}\right)\big|_{r=a} = \vec{r}\cdot\left(\vec{\nabla}\times\vec{u}(a,\theta,\varphi)\right)$$

is valid, because the projection of the curl in radial direction does not depend on derivatives with respect to r.

Now we need to relate the flow fields inside the sphere to the velocity field given on the surface. For this we can use another useful property of the solid harmonic functions: Their dependence on the radial coordinate is completely determined by their order:

$$f^n(r,\theta,\varphi)\big|_{r=a} = \left(\frac{a}{r}\right)^n f^n(r,\theta,\ \varphi).$$

Using this property, the fields χ, ϕ and p anywhere in the domain can be related to

the velocities given on the surface in the following way:

$$u_r(a,\theta,\varphi) = \sum_{n=-\infty}^{\infty}\left[\frac{n}{a}\frac{a^n}{r^n}\phi^n(r,\theta,\varphi) + \frac{n}{2\mu(2n+3)}a\frac{a^n}{r^n}p^n(r,\theta,\varphi)\right] \tag{17}$$

$$-r\vec{\nabla}\cdot\vec{u}(a,\theta,\varphi) = \sum_{n=-\infty}^{\infty}\left[\frac{n(n-1)}{a}\frac{a^n}{r^n}\phi^n(r,\theta,\varphi) + \frac{n(n+1)}{2\mu(2n+3)}a\frac{a^n}{r^n}p^n(r,\theta,\varphi)\right] \tag{18}$$

$$\vec{r}\cdot\left(\vec{\nabla}\times\vec{u}(a,\theta,\varphi)\right) = \sum_{n=-\infty}^{\infty}\left[n(n+1)\frac{a^n}{r^n}\chi^n(r,\theta,\varphi)\right] \tag{19}$$

At this point we need to make sure that our solution is well behaved in the domain of consideration. Normally, critical points are $r = 0$ and $r = \infty$. In this project, we are only interested in the domain inside the sphere, so any singularity at $r = \infty$ can be ignored. But singularities at $r = 0$ need to be avoided. Therefore we set all fields with negative order of n to 0:

$$\phi^n = \chi^n = p^n = 0, \qquad\qquad n < 0$$

A closer look at equation (13) reveals that even the terms with $n = 0$ do not contribute to the velocity field $\vec{u}$. Either solely the derivatives in the scalar fields matter (terms 1 to 3) or the prefactor $B = 0$ for $n = 0$. Therefore we have the freedom to set the $n = 0$ parts of the fields to 0:

$$\phi^n = \chi^n = p^n = 0. \qquad\qquad n < 1 \tag{20}$$

As the three functions on the left hand side of equations (17) to (19) are defined on the surface of the sphere and have their results in the reals they can be expanded in orders of spherical harmonic functions $X^n(\theta,\varphi), Y^n(\theta,\varphi), Z^n(\theta,\varphi)$ with:

$$u_r(a,\theta,\varphi) = \sum_{n=0}^{\infty} X^n(\theta,\varphi) \tag{21}$$

$$-r\vec{\nabla}\cdot\vec{u}(a,\theta,\varphi) = \sum_{n=0}^{\infty} Y^n(\theta,\varphi) \tag{22}$$

$$\vec{r}\cdot\left(\vec{\nabla}\times\vec{u}(a,\theta,\varphi)\right) = \sum_{n=0}^{\infty} Z^n(\theta,\varphi) \tag{23}$$

Combining equations (17)-(19) with (21)-(23), this results in:

$$\sum_{n=1}^{\infty} \frac{n}{a} \frac{a^n}{r^n} \phi^n(r,\theta,\varphi) + \frac{n}{2\mu(2n+3)} a \frac{a^n}{r^n} p^n(a,\theta,\varphi) = \sum_{n=0}^{\infty} X^n(\theta,\varphi)$$
$$\sum_{n=1}^{\infty} \frac{n(n-1)}{a} \frac{a^n}{r^n} \phi^n(r,\theta,\varphi) + \frac{n(n+1)}{2\mu(2n+3)} a \frac{a^n}{r^n} p^n(r,\theta,\varphi) = \sum_{n=0}^{\infty} Y^n(\theta,\varphi)$$
$$\sum_{n=1}^{\infty} n(n+1) \frac{a^n}{r^n} \chi^n(r,\theta,\varphi) = \sum_{n=0}^{\infty} Z^n(\theta,\varphi)$$

As the spherical harmonics make up a complete set of functions, any square integrable function on the sphere can be expanded in these. Since they are also orthogonal, each order of n must match separately. This allows us to solve these equations for the three fields of interest χ, ϕ and p that fully define the flows. This results in the following three equations for $n > 0$:

$$p^n(r,\theta,\varphi) = \frac{\mu(2n+3)}{na} \frac{r^n}{a^n} \left(Y^n - (n-1)X^n\right) \tag{24}$$
$$\phi^n(r,\theta,\varphi) = \frac{a}{2n} \frac{r^n}{a^n} \left((n+1) X^n - Y^n\right) \tag{25}$$
$$\chi^n(r,\theta,\varphi) = \frac{1}{n(n+1)} \frac{r^n}{a^n} Z^n. \tag{26}$$

Interestingly, the flow field from equation 13 now becomes independent of the viscosity μ, as the pressure field is divided by μ. Thus, the pressure field is the only field depending on viscosity μ. This becomes apparent when comparing it to the solution for shear flow or more specific plane Couette flow. In this case the viscosity of the fluid does not change the velocity profile, too. In contrast, the dissipated energy and thus the force needed to keep one wall moving, depends on viscosity.

To summarise, the only input we have to give are the velocities on the surface of the sphere $\vec{u}(a,\theta,\varphi)$. This velocity field and certain derivatives of it then have to be expanded in orders of spherical harmonic functions. These are recombined to three scalar fields, defined on the interior of the sphere. A combination of the scalar fields and their derivatives then gives the velocity field inside the whole sphere.

This is a major step in order to describe the flow field inside the oocyte but the method of solution is only valid for sphere-like boundaries. To first order the oocytes are sphere like but in this project we explicitly want to describe the deformation of the oocyte. Therefore we should also consider flows inside a deformed sphere. This can be achieved by following a perturbation theory ansatz.

Flows inside a Slightly Deformed Sphere

In this part we follow Brenner [3], who has developed a perturbation ansatz for flows outside of a slightly deformed sphere $\mathcal{S}_d$ and present it for the flows inside a deformed sphere with given velocity field on the surface. We assume that the deformation of the sphere should be of order $\varepsilon < 1$, meaning:

$$r(\theta, \varphi) = a\left(1 + \varepsilon f(\theta, \varphi)\right), \tag{27}$$

where $f(\theta, \varphi) = \sum_{n=0}^{\infty} S^n(\theta, \varphi)$ is a linear combination of spherical harmonics. These radii $r(\theta, \varphi)$ define the boundary of our object of consideration $\mathcal{S}_d$. Next we assume, that also the velocity field $\vec{u}$ and the pressure field p can be expanded in orders of ε:

$$\vec{u} = \sum_{k=0}^{\infty} \varepsilon^k \vec{u}^{(k)}, \qquad p = \sum_{k=0}^{\infty} \varepsilon^k p^{(k)}.$$

Plugging these into the inhomogeneous Stokes equation, we see that the solutions must match order by order:

$$\vec{\nabla}^2 u_i^{(k)} = \mu \frac{\partial p^{(k)}}{\partial x_i}$$
$$\vec{\nabla} \cdot \vec{u}^{(k)} = 0.$$

In principle, we know how to solve these equations as shown in the previous chapter. The only thing we still need to do is to match the boundary conditions for each order in ε. We set $\vec{U}(\theta, \varphi)$ to be the velocity field on the surface $\mathcal{S}_d$. Using the notation from the previous chapter this can be written as: $\vec{U}(\theta, \varphi) = \vec{u}(r(\theta, \varphi), \theta, \varphi)$. The no-slip boundary condition on the surface consequently reads:

$$\vec{u} = \sum_{k=0}^{\infty} \varepsilon^k \vec{u}^{(k)} = \vec{U}(\theta, \varphi), \qquad \text{on } \mathcal{S}_d$$

As we are looking at the flows inside the surface, there is no need to match up any further boundary condition.

Each order of $\vec{u}^{(k)}$ can be expanded by a Taylor expansion on its own around the undeformed boundary with $r = a$:

$$\vec{u}^{(k)} = \vec{u}^{(k)}\Big|_{r=a} + \sum_{j=1}^{\infty} \frac{(r-a)^j}{j!} \frac{\partial^j \vec{u}^{(k)}}{\partial r^j}\Bigg|_{r=a}$$

Plugging in the expression for r (27) we get:

$$\vec{u}^{(k)} = \vec{u}^{(k)}\big|_{r=a} + \sum_{j=1}^{\infty} \frac{(\varepsilon a f(\theta,\varphi))^j}{j!} \frac{\partial^j \vec{u}^{(k)}}{\partial r^j}\bigg|_{r=a}$$

summing over all orders of k and after grouping orders of ε we end up with the condition:

$$\vec{U}(\theta,\varphi) = \vec{u}^{(0)}\big|_{r=a} + \sum_{k=1}^{\infty} \varepsilon^k \left(\vec{u}^{(k)}\big|_{r=a} + \sum_{j=1}^{k} \frac{(a f(\theta,\varphi))^j}{j!} \frac{\partial^j \vec{u}^{(k-j)}}{\partial r^j}\bigg|_{r=a} \right) \tag{28}$$

To make it most convenient, we choose the following scheme of solution:

$$\vec{u}^{(0)}\big|_{r=a} = \vec{U}(\theta,\varphi)$$

and for $k > 0$

$$\varepsilon^k \left(\vec{u}^{(k)}\big|_{r=a} + \sum_{j=1}^{k} \frac{(a f(\theta,\varphi))^j}{j!} \frac{\partial^j \vec{u}^{(k-j)}}{\partial r^j}\bigg|_{r=a} \right) = 0.$$

This can be rewritten to:

$$\vec{u}^{(k)}\big|_{r=a} = -\sum_{j=1}^{k} \frac{(a f(\theta,\varphi))^j}{j!} \frac{\partial^j \vec{u}^{(k-j)}}{\partial r^j}\bigg|_{r=a} \tag{29}$$

Explicitly this reads:

$$\vec{u}^{(0)}\big|_{r=a} = \vec{U}(\theta,\varphi) \tag{30}$$

$$\vec{u}^{(1)}\big|_{r=a} = -a f(\theta,\varphi) \frac{\partial \vec{u}^{(0)}}{\partial r}\bigg|_{r=a} \tag{31}$$

$$\vec{u}^{(2)}\big|_{r=a} = -a f(\theta,\varphi) \frac{\partial \vec{u}^{(1)}}{\partial r}\bigg|_{r=a} - \frac{a^2}{2} f^2(\theta,\varphi) \frac{\partial^2 \vec{u}^{(0)}}{\partial r^2}\bigg|_{r=a} \tag{32}$$

$$\dots .$$

In principle this can be expanded to arbitrarily high order but the amount of terms becomes more and more difficult to handle. We have to admit that there is no statement of convergence of this perturbation scheme. As the parameter ε only appears in terms like $\varepsilon f(\theta,\varphi)$ the convergence does not depend on the choice of ε. This clearly shows

that the hope for convergence depends on the combined term $-1 \ll \varepsilon f(\theta,\varphi) \ll 1$. In equation (29) it is shown that for each higher order in perturbation only the lower orders are needed. Explicitly the following path can be taken: At first the unperturbed flow field $\vec{u}^{(0)}$ is calculated as described for the unperturbed flows using the boundary flows $\vec{u}^{(0)}\big|_{r=a} = \vec{U}(\theta,\varphi)$. For the boundary flows $\vec{U}$ we use the ones from the perturbed surface. Next, the solution is plugged into equation (31) and the boundary flows $\vec{u}^{(1)}\big|_{r=a}$ are obtained. After this, the procedure is repeated. At this point, the boundary flows $\vec{u}^{(1)}\big|_{r=a}$ are used to calculate the full first order solution $\vec{u}^{(1)}$. The solution for the full domain can be calculated as given before, as each order on its own has to obey the Stokes equation. This procedure can be repeated for all higher orders. The flow inside the perturbed boundary then results in the sum of the individual orders

$$\vec{u} = \sum_{k=0}^{\infty} \varepsilon^k \vec{u}^{(k)}.$$

Detailed Curvature Calculation

We start with a rotationally symmetric 2D surface embedded in 3D space given by:

$$\vec{f}(\varphi,\theta) = R(\theta)\begin{pmatrix}\cos\theta\cos\varphi\\ \cos\theta\sin\varphi\\ \sin\theta\end{pmatrix}. \tag{33}$$

Here $\theta \in \left[-\frac{\pi}{2},\frac{\pi}{2}\right)$ is the polar angle measured from xy-plane and $\varphi \in [0,2\pi)$ is the azimuthal angle measured from xz-plane. Note the definition of the polar coordinates, which here is not defined in the range $[0,\pi)$. This surface is independent of φ showing the axisymmetry of the setup.

Mean Curvature H and Gaussian Curvature K can be calculated as the determinant and trace of the Weingarten matrix a:

$$H = \frac{1}{2}\mathrm{tr}\; a, \qquad\qquad K = \det a. \tag{34}$$

The Weingarten matrix can be calculated as

$$\mathbf{a} = \mathbf{h}\mathbf{g}^{-1}, \tag{35}$$

with g^{-1} being the inverse of the metric tensor or first fundamental form and h the second fundamental form. The metric tensor can be computed as

$$g_{ij} = \partial_i \vec{f} \cdot \partial_j \vec{f}. \tag{36}$$

In the specific case of rotational symmetry and considering φ as first and θ as second coordinate $\mathbf{g}$ takes the following form:

$$\mathbf{g} = \begin{pmatrix} R^2 \cos^2\theta & 0 \\ 0 & R^2 + (\partial_\theta R)^2 \end{pmatrix} \tag{37}$$

and its determinant:

$$\det \mathbf{g} = R^2 \cos^2\theta \left(R^2 + (\partial_\theta R)^2 \right).$$

For the second fundamental form we know that it has the form:

$$h_{ij} = -\partial_i \vec{n} \cdot \partial_j \vec{f} = \vec{n} \partial_i \partial_j \vec{f}, \tag{38}$$

where we used for the second equality that the normal vector $\vec{n}$ is perpendicular to the tangent vectors $\partial_i \vec{f}$. Additionally the product rule for differentiation was applied.

For the normal vector $\vec{n}$ we obtain:

$$\vec{n} = \frac{\partial_\varphi \vec{f} \times \partial_\theta \vec{f}}{\left|\partial_\varphi \vec{f} \times \partial_\theta \vec{f}\right|} = \frac{\vec{f} + \partial_\theta R \begin{pmatrix} \sin\theta\cos\varphi \\ \sin\theta\sin\varphi \\ \cos\theta \end{pmatrix}}{\sqrt{R^2 + (\partial_\theta R)^2}}. \tag{39}$$

Plugging these results into equation (38) we obtain:

$$\mathbf{h} = \frac{1}{\sqrt{R^2 + (\partial_\theta R)^2}} \left[-\mathbf{g} + \begin{pmatrix} -R\,(\partial_\theta R) \sin\ \theta\cos\theta & 0 \\ 0 & -(\partial_\theta R)^2 + R\,(\partial_\theta \partial_\theta R) \end{pmatrix} \right]. \tag{40}$$

For the inverse of $\mathbf{g}$, being a diagonal two by two matrix, we obtain:

$$\mathbf{g}^{-1} = \frac{1}{\det \mathbf{g}} \begin{pmatrix} g_{22} & 0 \\ 0 & g_{11} \end{pmatrix}.$$

As $\mathbf{g}$ and $\mathbf{h}$ are both diagonal, also the Weingarten matrix $\mathbf{a}$ is diagonal. This then

leads to the two principal curvatures a_{11} and a_{22}:

$$a_{\varphi\varphi} = h_{\varphi\varphi}\left(g^{-1}\right)_{\varphi\varphi} = \frac{-1 - \frac{\left(R^2+(\partial_\theta R)^2\right)R(\partial_\theta R)\sin\ \theta\cos\theta}{R^2\cos^2\theta\left(R^2+(\partial_\theta R)^2\right)}}{\sqrt{R^2+(\partial_\theta R)^2}} = \frac{-1 - \frac{(\partial_\theta R)\tan\theta}{R}}{\sqrt{R^2+(\partial_\theta R)^2}}, \quad (41)$$

$$a_{\theta\theta} = h_{\theta\theta}\left(g^{-1}\right)_{\theta\theta} = \frac{-1 + \frac{R^2\cos^2\theta\left(-(\partial_\theta R)^2+R(\partial_\theta\partial_\theta R)\right)}{R^2\cos^2\theta\left(R^2+(\partial_\theta R)^2\right)}}{\sqrt{R^2+(\partial_\theta R)^2}} = \frac{-1 + \frac{\left(-(\partial_\theta R)^2+R(\partial_\theta\partial_\theta R)\right)}{\left(R^2+(\partial_\theta R)^2\right)}}{\sqrt{R^2+(\partial_\theta R)^2}}. \quad (42)$$

From these mean curvature (H) and Gaussian curvature (K) are computed as:

$$H = \frac{a_{\varphi\varphi} + a_{\theta\theta}}{2}, \qquad K = a_{\varphi\varphi}a_{\theta\theta}. \quad (43)$$

Thus, the two principal curvatures are the curvatures measured in the direction of the coordinate axes, so the in-plane curvature measured is simply the curvature in θ direction $K_\theta := a_{\theta\theta}$.

Clearly, if one plugs in the surface of a sphere with $R(\theta) = \mathbf{const}$ all derivatives drop out and we obtain the correct result of $-\frac{1}{R}$ for the curvature at all points in all directions. Additionally, one observes the divergence of $a_{\varphi\varphi}$ at the poles where $\theta = -\frac{\pi}{2}$ and $\theta = \frac{\pi}{2}$ if $\partial_\theta R$ does not vanish there. But what does this mean? This means that the surface is not flat at $\theta = 0$ but pointed, like a cone. Thus, of course the curvature in φ direction becomes infinite at this point, as the radius of the cone goes to zero.

References

1. Horace Lamb. *Hydrodynamics.* 6. ed. Cambridge: Univ. Pr., 1975, XV, 738 S. ISBN: 0-521-05515-6.

2. John Happel and Howard Brenner. *Low Reynolds number hydrodynamics. with special applications to particulate media.* eng. Second revised ed, first paperbackack edition. Mechanics of fluids and transport processes. Originally published: Leiden : Noordhoff, 1973. - Includes index. The Hague ; Boston ; Lancaster: Martinus Nijhoff Publishers, 1983, p. 553. ISBN: 978-94-009-8352-6.

3. Howard Brenner. “The Stokes resistance of a slightly deformed sphere”. In: *Chemical Engineering Science* 19.8 (1964), pp. 519–539. DOI: 10.1016/0009-2509(64)85045-4. URL: https://doi.org/10.1016/0009-2509(64)85045-4.
